# Climatic Adaptability Changes in Leaf Functional Traits of Old *Pinus tabulaeformis* in Loess Plateau

**DOI:** 10.3390/plants14142128

**Published:** 2025-07-10

**Authors:** Yuting Lei, Zimao Feng, Zhong Zhao

**Affiliations:** 1 Key Comprehensive Laboratory of Forestry, Northwest A&F University, Yangling 712100, China; leiytg@nwafu.edu.cn (Y.L.); fengzimao@nwafu.edu.cn (Z.F.); 2 Key Laboratory of Silviculture on the Loess Plateau State Forestry Administration, Northwest A&F University, Yangling 712100, China

**Keywords:** arid and semi-arid areas, conservation of old trees, geographical pattern, ecological adaptation, traits variation

## Abstract

A systematic examination of leaf functional traits, environmental determinants, and adaptive regulation strategies in old *Pinus tabuliformis* was conducted in the Loess Plateau region. During the peak growth period (July) of *P. tabuliformis* in 2023 and 2024, integrating phylogenetic comparative methods with environmental gradient analysis, we quantified 28 functional traits (7 morphological, 8 anatomical, 5 chemical, and 8 physiological traits) of old *P. tabuliformis*. The result shows significant spatial differentiation in leaf chemical and physiological traits, demonstrating exceptional environmental plasticity. Old trees in the Huanglong area of central China tend to be of the resource acquisition type, while the proportion of the Stress-tolerators strategy (S strategy) is higher in the Taibai (S% = 92.32). The combined effect of environmental factors is the main driving factor for the diversity of leaf functional traits (33.56%), while the independent effect of phylogenetic accounts for only 8.91%. And regression modeling identified several traits, such as Malondialdehyde (MDA), Peroxidase (POD), and Superoxide dismutase (SOD), as sensitive indicators of geographical and climatic adaptation. In conclusion, this study elucidates drought adaptation mechanisms in old *P. tabuliformis* through leaf functional trait analysis, establishing a scientific framework for conserving old trees in Loess Plateau under climate change.

## 1. Introduction

Old trees that have reached a century or more in age represent irreplaceable conservation resources due to their profound ecological, evolutionary, and cultural value [1]. From an ecological perspective, the oldest living trees serve not only as invaluable historical benchmarks for investigating forest growth sensitivity to climate variability and long-term dynamics under climatic constraints over millennia [2,3], but also across numerous global ecosystems, geographically dispersed or small clusters of old trees still persist as vital contributors to carbon sequestration and providing wildlife habitat [4]. Within evolutionary frameworks, old trees are pivotal contributors in species-level viability, buffer genome, and resilience against multi-generational environmental shifts, and are indispensable subjects for investigating botanical adaptation over extended temporal scales [5]. However, diminishing populations of old trees has been hastened by amplified anthropogenic pressures over extended periods, such as increasing temperatures and carbon dioxide (CO_2_) concentrations, coupled with recurring episodic disruptions like wildfires, droughts, pest infestations, and land-use changes [6,7]. With increasing recognition of the ecological significance of old trees, diverse conservation measures have been proposed and implemented worldwide. For instance, regions such as England and Sydney have established specific policies aimed at enhancing the protection of large ancient trees [8]. Notably, China has strengthened these conservation efforts through key national legislation, including the Forest Law of the People’s Republic of China and the Criminal Law of the People’s Republic of China [9]. However, policies implemented at a single jurisdictional level remain insufficient to adequately safeguard large old trees. Moreover, scientific understanding of how environmental changes affect their growth and physiological processes remains limited. Effective conservation policies and management strategies require scientific understanding of old trees, which underscores the necessity of dedicated research to them.

Leaf functional traits constitute essential proxies of vegetative development and ecological adaptation strategies [10]. They regulate fundamental ecological processes, such as gas exchange, carbon sequestration, photosynthesis, and are the key to elucidating plant coordination and adaptation mechanisms [11]. Consequently, in contemporary ecological studies they have garnered sustained scholarly attention [12,13,14]. Leaf functional traits typically include morphological, anatomical, chemical, and physiological traits. Leaf morphological traits significantly influence adaptive and developmental potential under diverse environmental conditions [15], for instance, the “leaf economics spectrum” suggests that leaf thickness, leaf area, and leaf mass per area may reflect the photosynthetic capacity of plants at broad geographical scales [11]. Anatomical traits, primarily comprising stomata, mesophyll, and vascular tissues, underpin both leaf morphological variation and diverse physiological and biochemical processes [16]. Stomata and epidermal tissues fundamentally regulate hydric equilibrium and defense mechanisms [17,18], while vascular tissues ensure structural integrity, facilitate solute translocation and indicate carbon allocation within the plant vascular system [19]. Chemical traits based on mineral elements reflect the survival mode and nutrient utilization characteristics of plants [20]. As an illustration, nitrogen and phosphorus serve as essential nutrients that regulate development, metabolic functions, and overall growth [21]; stoichiometric ratios serve as indicators to assess environmental nutrient availability and signal potential nutrient limitations [22]. Leaf water content, osmotic regulatory substances, and antioxidant enzyme activity are standard indices for evaluating plant physiological traits due to their sensitivity to short-term environmental changes and their capacity to quantify environmental stress on plants [23]. Leaf functional traits have emerged as a key research focus across multiple disciplines, and numerous studies have been conducted on *P. tabuliformis*. Su et al. (2021) analyzed leaf functional traits of *P. tabuliformis* across three land-use types in urban and rural areas of Beijing, revealing significant variations among different leaf ages and land-use types [24]; Zhang et al. (2023) reported in their Loess Plateau study that *P. tabuliformis* adapts to drought conditions by modulating leaf anatomical, photosynthetic, and biochemical traits [25]. However, existing studies predominantly focus on young or mature plants, with limited attention to old trees. Pasques and Munné-Bosch’s (2024) study found that old trees have unique evolutionary characteristics (adaptability, modular autonomy, etc.) that allow them to develop complex morphological, biochemical, and physiological adaptations, such as improved stress resistance and regenerative capacities that prioritize survival over biomass accumulation, resulting in extraordinary longevity [26]. Consequently, analyzing leaf functional traits of old trees is critical to understanding their ecological resilience. Furthermore, recent studies have increasingly recognized the limitations of relying on only one or a few plant traits to assess [27]. A comprehensive multi-trait approach, integrating morphological, anatomical, chemical, and physiological traits, provides a robust framework to elucidate how old trees adapt to environmental pressures.

Environmental factors, including climatic, edaphic, and geographical factors, exert considerable influence on the diversity of leaf functional traits. In recent decades, extensive research has examined distributional configurations of leaf traits along continental environmental gradients [28,29], demonstrating that environmental factors predominantly drive trait variability [30]. Subsequent research indicates that global-scale trait variation is primarily governed by the combined influence of climatic and edaphic conditions, and additional independent climate effects have been observed in most traits [31]. However, Zhang et al. (2023) argue that within the Loess Plateau, edaphic factors—rather than climatic variables—directly control leaf functional traits variation [25]. These conflicting findings underscore the need for context-specific analyses to clarify environmental drivers of leaf functional traits. Furthermore, investigations of *Alnus nepalensis* across ontogenetic stages reveal age-dependent shifts in leaf traits [32]. Whether environmental influences on old trees align with trends observed in broader studies remains uncertain. Similarly, phylogeny is also considered to have an important influence on leaf traits variation [33]. More and more studies have been conducted to analyze synergistic or coupling relationships among traits and their environmental adaptability in the context of species phylogeny [34]. For instance, phylogeny explained 3.9–23.3% variation in leaf traits in 1819 plants from China [35]. While phylogenetic analysis is mostly applied to interspecific comparisons, they are increasingly leveraged for intraspecific analyses, particularly in taxa with undocumented provenance or geographically distinct ecotypes. For example, Wu et al. (2022) reported strong phylogenetic signals in foliar elongation and propagule weight among *Phragmites australis* populations in coastal wetland ecosystems of the Huanghe Estuary [36]. By integrating the evolutionary history of species, phylogenetic information can provide useful insights into interpreting the adaptive and functional significance of plant traits.

Located in arid and semi-arid regions, the Loess Plateau is characterized by its distinct loess geology, fragmented geomorphology, and fragile hydrological environment, and is defined as a “difficult site” by traditional forest cultivation theory [37]. *Pinus tabuliformis*, the dominant afforestation species in the area, exhibits pronounced cold and drought tolerance [38]. However, persistent climate change and intensifying anthropogenic disturbances have led to the decline of the number of old *P. tabuliformis*, leaving only fragmented remnants in marginal habitats such as ridges, where environmental conditions are highly unfavorable. At present, the extant old *P. tabuliformis* resources in China are primarily distributed in Beijing, Shandong, Shaanxi, and Hebei provinces. This study investigates long-term survival adaptations in old *P. tabuliformis* through systematic analysis of leaf functional traits, providing critical insights for designing targeted preservation protocols. This research examined foliar characteristics and environmental conditions associated with old *P. tabuliformis* across three distinct regions of the Loess Plateau, addressing three objectives: (1) characterizing geographical differentiation in leaf functional traits among old *P. tabuliformis* while identifying synergies or trade-offs among them; (2) determining the primary drivers and mechanisms underlying trait variability; and (3) identifying traits most sensitive to environmental changes.

## 2. Results

### 2.1. Characteristics and Variation in Leaf Variation of Leaf Functional Traits in Different Regions

**Figure 1:**
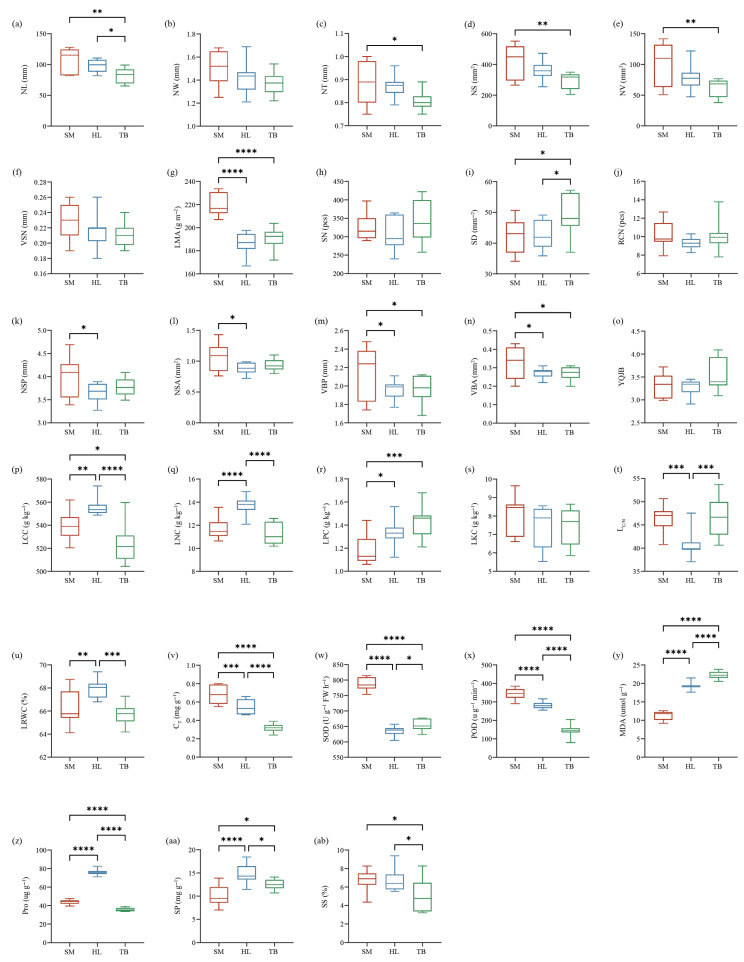
Box map of (**a**–**g**) morphological traits, (**h**–**o**) anatomical traits, (**p**–**t**) chemical traits, and (**u**–**ab**) physiological traits. * means *p* < 0.05; ** means *p* < 0.01; *** means *p* < 0.001; **** means *p* < 0.0001. NL, needle length; NW, needle width; NT, needle thickness; NS, needle surface area; NV, needle volume; VSN, the ratio of NV to NS; LMA, leaf mass per area; SN, stomata number; SD, stomatal density; RCN, resin canal number; NSP, needle section perimeter; NSA, needle section area; VBP, vascular bundle perimeter; VBA, vascular bundle area; YQJB, the ratio of NSA to VBA; LCC, contents of carbon; LNC, contents of nitrogen; LPC, contents of phosphorus; LKC, contents of potassium; L_C/N_, carbon nitrogen ratio; LRWC, leaf water content; C_T_, total chlorophyll content; SOD, superoxide dismutase activity; POD, peroxidase activity; MDA, malondialdehyde content; Pro, proline content; SP, soluble protein content; SS, soluble sugar content.

Geographical variation patterns were identified through an assessment of 28 functional leaf traits in old *P. tabuliformis* (Figure 1). One-way ANOVA demonstrated significant interregional differences in most chemical and physiological traits (*p* < 0.05, except LKC), while morphological and anatomical traits displayed limited regional differentiation. CV of leaf functional traits showed significant differences among all old *P. tabuliformis* (Table 1), with the highest NV (34.55%) and the lowest LRWC (2.07%).

### 2.2. Correlation of Leaf Functional Traits

**Figure 2:**
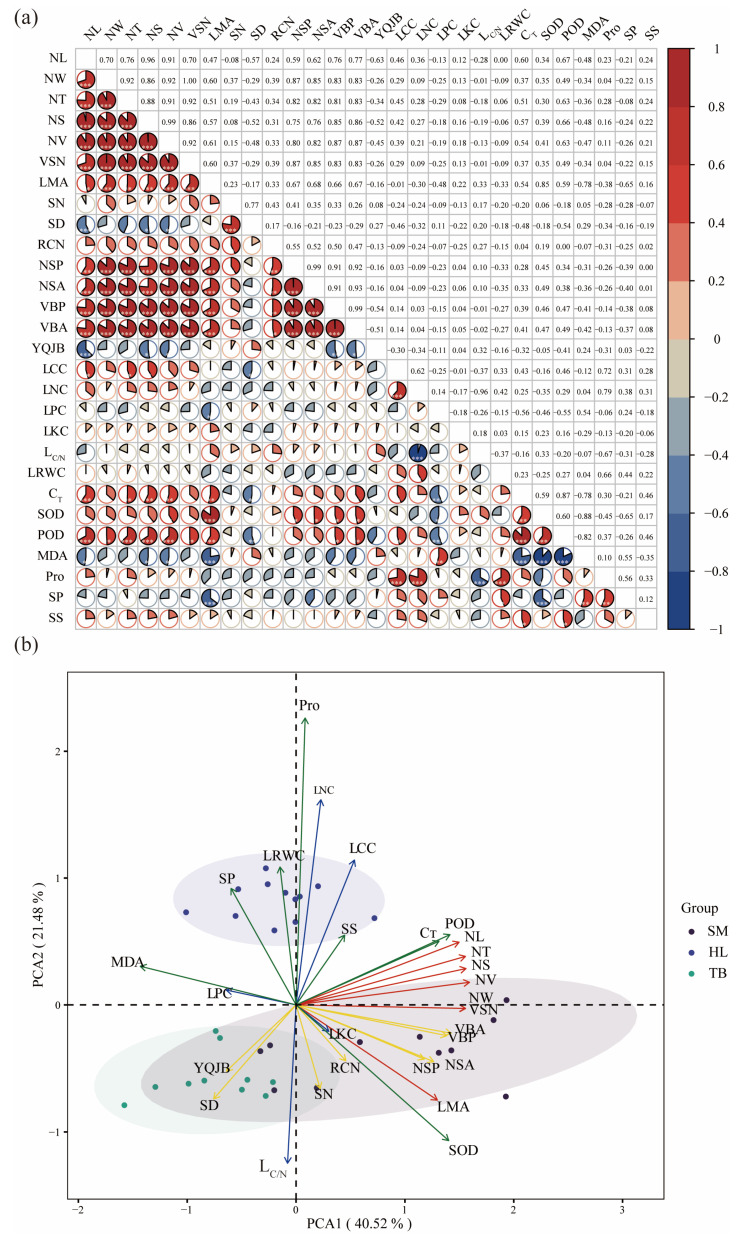
(**a**) Correlation heat map and (**b**) PCA analysis of leaf functional traits of old trees. *** *p* < 0.001; ** *p* < 0.01; * *p* < 0.05. NL, needle length; NW, needle width; NT, needle thickness; NS, needle surface area; NV, needle volume; VSN, the ratio of NV to NS; LMA, leaf mass per area; SN, stomata number; SD, stomatal density; RCN, resin canal number; NSP, needle section perimeter; NSA, needle section area; VBP, vascular bundle perimeter; VBA, vascular bundle area; YQJB, the ratio of NSA to VBA; LCC, contents of carbon; LNC, contents of nitrogen; LPC, contents of phosphorus; LKC, contents of potassium; L_C/N_, carbon nitrogen ratio; LRWC, leaf water content; C_T_, total chlorophyll content; SOD, superoxide dismutase activity; POD, peroxidase activity; MDA, malondialdehyde content; Pro, proline content; SP, soluble protein content; SS, soluble sugar content.

Distinct modular patterns were observed in the marked associations between leaf functional traits. Anatomical traits (except stomata-related traits) demonstrated strong positive associations with morphological traits (*p* < 0.05). Some physiological traits (C_T_, SOD, POD, and MDA) were significantly correlated with morphological and anatomical traits (*p* < 0.05). In contrast, chemical traits displayed limited correlation with other traits, and were only significantly correlated with MDA and Pro, for example, Pro was positively correlated with LCC and LNC (*p* < 0.001) (Figure 2a).

PCA revealed distinct covariation patterns in leaf functional traits of old *P. tabuliformis* (Figure 2b). The first two principal components (PC1 and PC2) cumulatively explained 62.00% of total variance (PC1 = 40.52%, PC2 = 21.48%). The morphological and anatomical traits were predominantly loaded on PC1 (positive quadrant), with morphological traits exhibiting relatively higher loading values. Specifically, NV, NW, VSN, NT, and NL showed significant loadings on this component. In contrast, chemical and physiological traits were primarily associated with PC2, where Pro, SOD, LRWC, L_C/N_, and LCC demonstrated prominent loading values. Notably, L_C/N_ and SOD displayed inverse relationships with other traits in this component. The old trees of Huanglong and the other two places were separated on the PC2 axis, while the old trees of Shenmu and Taibai exhibited overlapping distributions.

### 2.3. Phylogenetic Signal Analysis

Genetic variation in *P. tabuliformis* in different regions was studied by SSRs. Significant heterogeneity in genetic diversity was observed among regions. PCA demonstrated limited resolution, failing to separate old *P. tabuliformis* in three regions (Figure S1a). Nei’s genetic distance clustering revealed distinct regional groupings at a threshold of D = 0.030 (Figure S1b). Among them, Shenmu and Taibai old trees had a close genetic distance, while Huanglong and the two were relatively independent and had long genetic distance.

To assess phylogenetic signals in trait evolution, we employed two complementary metrics: Blomberg’s *K*, which quantifies the strength of phylogenetic signal by comparing the observed variance of traits to that expected under a Brownian motion model; and Pagel’s λ, a branch-length transformation parameter that evaluates trait evolution patterns. Among 28 leaf functional traits analyzed (Table 2), Blomberg’s *K* values ranged 0.66–1.08 (mean = 0.81, RCN is the smallest, LCC is the largest), and Pagel’s λ estimates varied 0–1 (mean = 0.28). Except for SD, LCC, LNC, LRWC, and Pro, which showed significant phylogenetic signals (*p* < 0.05), the other traits all exhibited weak phylogenetic signals and were not statistically significant (*p* > 0.05). Collectively, these functional traits demonstrate attenuated phylogenetic conservatism, suggesting that habitat filtering supersedes evolutionary constraints in shaping leaf traits.

### 2.4. Environmental Driving Factors of Leaf Functional Traits of Old Tree

**Figure 3:**
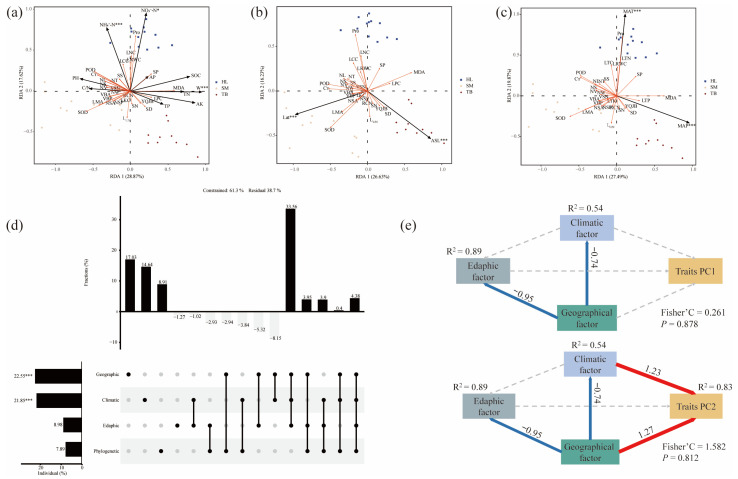
Analysis of driving factors of leaf functional traits. RDA ranking of leaf functional traits by (**a**) edaphic factors, (**b**) geographical factors, and (**c**) climatic factors. (**e**) Hierarchical segmentation analysis of environmental factors. (**d**) SEM examined the direct or indirect effects of environmental variables on leaf functional traits. *** *p* < 0.001; * *p* < 0.05. NL, needle length; NW, needle width; NT, needle thickness; NS, needle surface area; NV, needle volume; VSN, the ratio of NV to NS; LMA, leaf mass per area; SN, stomata number; SD, stomatal density; RCN, resin canal number; NSP, needle section perimeter; NSA, needle section area; VBP, vascular bundle perimeter; VBA, vascular bundle area; YQJB, the ratio of NSA to VBA; LCC, contents of carbon; LNC, contents of nitrogen; LPC, contents of phosphorus; LKC, contents of potassium; L_C/N_, carbon nitrogen ratio; LRWC, leaf water content; C_T_, total chlorophyll content; SOD, superoxide dismutase activity; POD, peroxidase activity; MDA, malondialdehyde content; Pro, proline content; SP, soluble protein content; SS, soluble sugar content.

This study examines the influence of three key environmental factors—edaphic, geographical, and climatic—on trait variation, independent of evolutionary processes. First, RDA was employed to independently assess the effects of each environmental factor. For edaphic factors, the first two RDA axes explained 46.49% of the observed variation, with W, NH_3_^−^-N, and NH_4_^+^-N identified as the primary drivers significantly associated with leaf functional trait variation (*p* < 0.05; Figure 3a). Similarly, geographical factors explained 42.86% of the variation, predominantly driven by Lat and ASL (*p* < 0.05; Figure 3b). Climatic factors explained 47.36% of the variation, with all climatic variables significantly influencing leaf functional traits of old *P. tabuliformis* (*p* < 0.05; Figure 3c). Secondly, hierarchical segmentation was used to explore the relative contribution degrees of environmental factors and phylogeny to trait variations (Figure 3d). The results showed that the comprehensive influence of the three environmental factors on the functional traits of leaves was the greatest (33.56%). The independent influences of geographical factors, climatic factors, and phylogenetic factors were 17.03%, 14.64%, and 8.91%, respectively. Soil factors did not show obvious individual effects and only acted through the synergistic interaction with other environmental variables. Finally, SEM was employed to evaluate the direct and indirect effects of environmental factors on leaf functional traits (PC1 and PC2). The analysis revealed (Figure 3e) that PC1 exhibited no direct influence from environmental factors, whereas PC2 was positively influenced by geographical and climatic factors, with potential indirect mediation by edaphic factors.

### 2.5. CSR Survival Strategies

**Figure 4:**
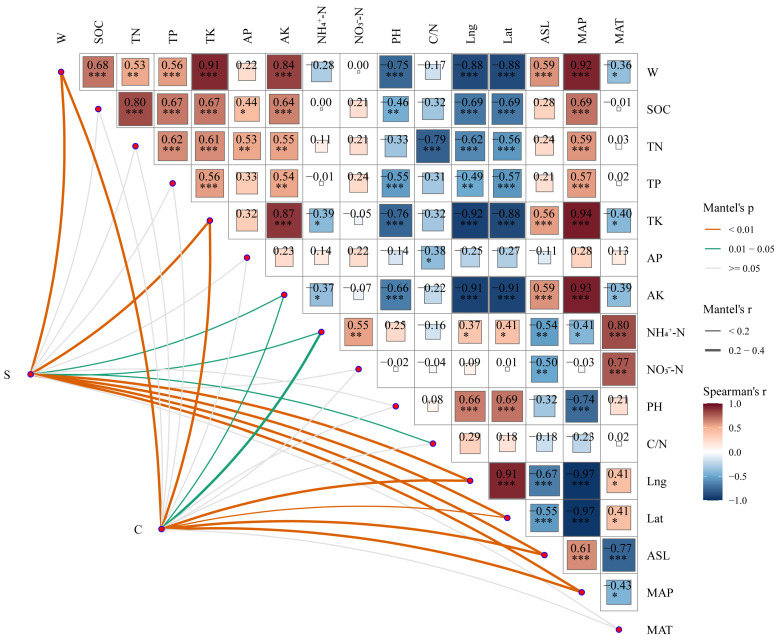
Mantel analysis of CSR strategies and environmental factors. *** *p* < 0.001; ** *p* < 0.01; * *p* < 0.05.

Although *P. tabuliformis* is typically characterized as an S-strategist species, our analysis revealed significant regional differences in C-, S-, and R-strategy values among old trees (Table 3). Notably, the S-strategy value in the Taibai population was significantly higher than those in Shenmu and Huanglong populations, while its C-strategy value was significantly lower compared to the other two regions (*p* < 0.05). These results indicate that the competitive intensity and pattern of resource utilization among old trees have shifted in the Taibai region, and the survival strategy has become relatively more conservative. Mantel’s tests demonstrated significant correlations between CSR strategy shifts and environmental variables, with W, TK, NH_4_^+^-N, and ASL identified as key driving factors (Figure 4).

### 2.6. Responses of Leaf Functional Traits to Geographical and Climatic Factors

**Figure 5:**
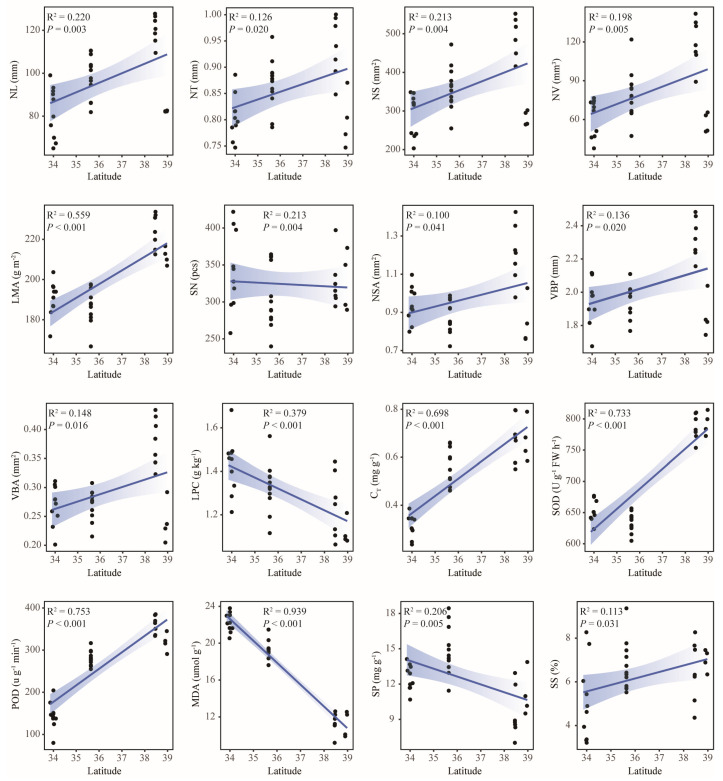
Linear regression analysis between leaf functional traits and latitude. NL, needle length; NT, needle thickness; NS, needle surface area; NV, needle volume; LMA, leaf mass per area; SN, stomata number; NSA, needle section area; VBP, vascular bundle perimeter; VBA, vascular bundle area; LPC, contents of phosphorus; C_T_, total chlorophyll content; SOD, superoxide dismutase activity; POD, peroxidase activity; MDA, malondialdehyde content; SP, soluble protein content; SS, soluble sugar content.

**Figure 6:**
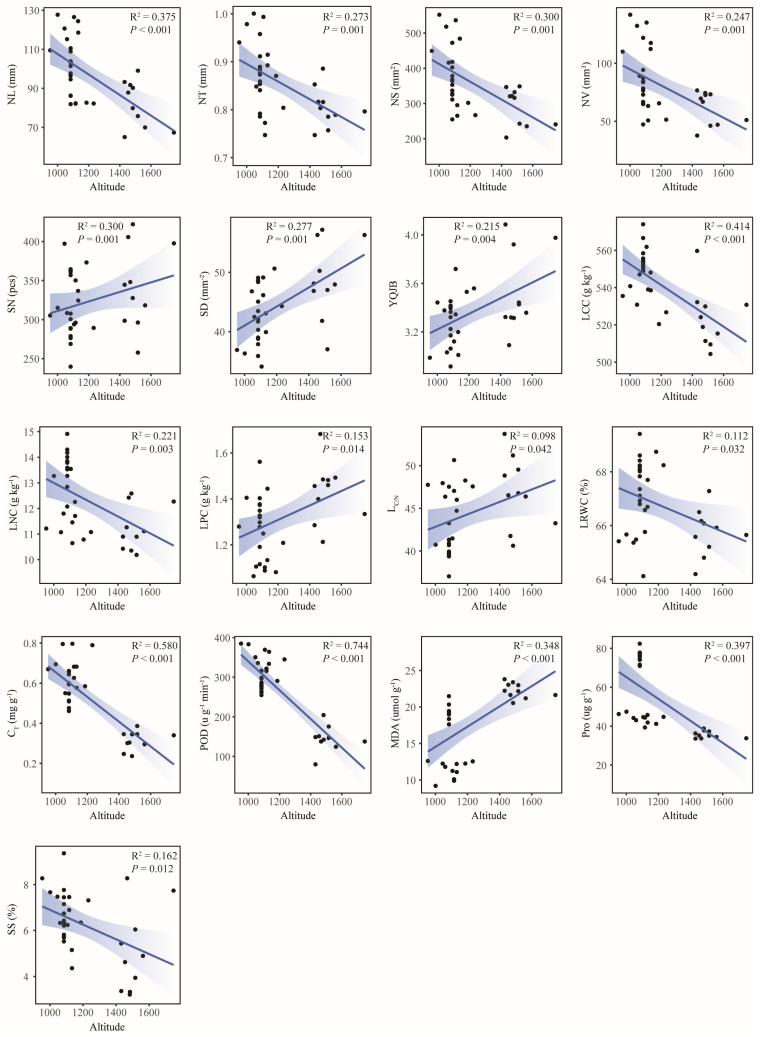
Linear regression analysis between leaf functional traits and altitude. NL, needle length; NT, needle thickness; NS, needle surface area; NV, needle volume; SN, stomata number; SD, stomatal density; YQJB, the ratio of NSA to VBA; LCC, contents of carbon; LNC, contents of nitrogen; LPC, contents of phosphorus; L_C/N_, carbon nitrogen ratio; LRWC, leaf water content; C_T_, total chlorophyll content; POD, peroxidase activity; MDA, malondialdehyde content; Pro, proline content; SS, soluble sugar content.

**Figure 7:**
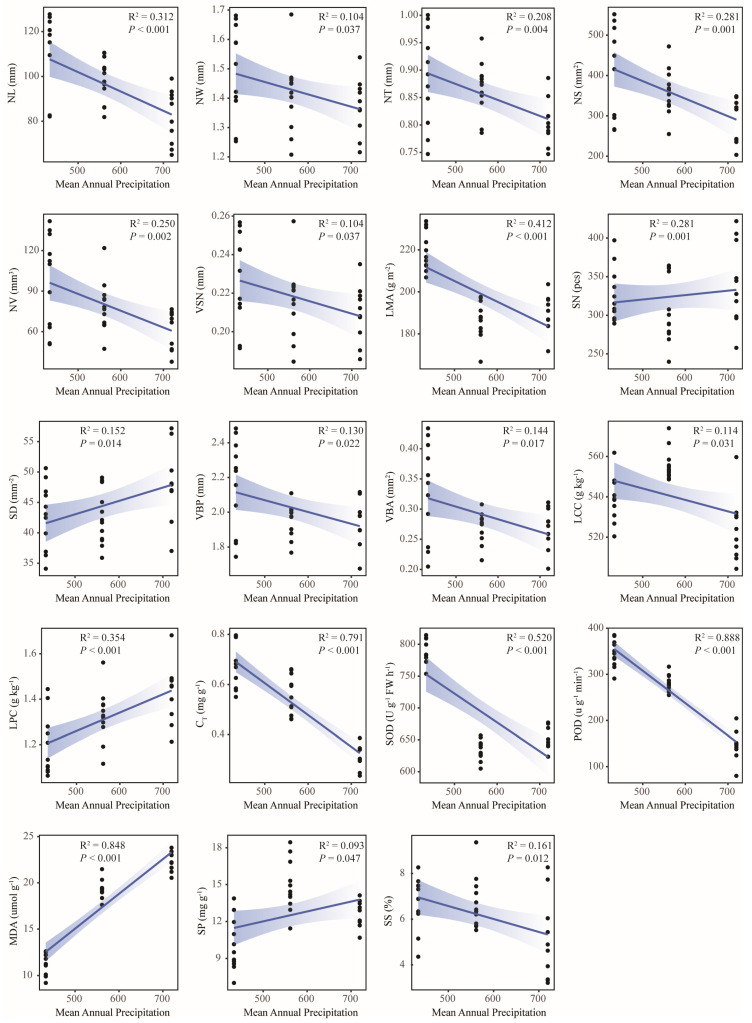
Linear regression analysis between leaf functional traits and mean annual precipitation. NL, needle length; NW, needle width; NT, needle thickness; NS, needle surface area; NV, needle volume; VSN, the ratio of NV to NS; LMA, leaf mass per area; SN, stomata number; SD, stomatal density; VBP, vascular bundle perimeter; VBA, vascular bundle area; LCC, contents of carbon; LPC, contents of phosphorus; C_T_, total chlorophyll content; SOD, superoxide dismutase activity; POD, peroxidase activity; MDA, malondialdehyde content; SP, soluble protein content; SS, soluble sugar content.

**Figure 8:**
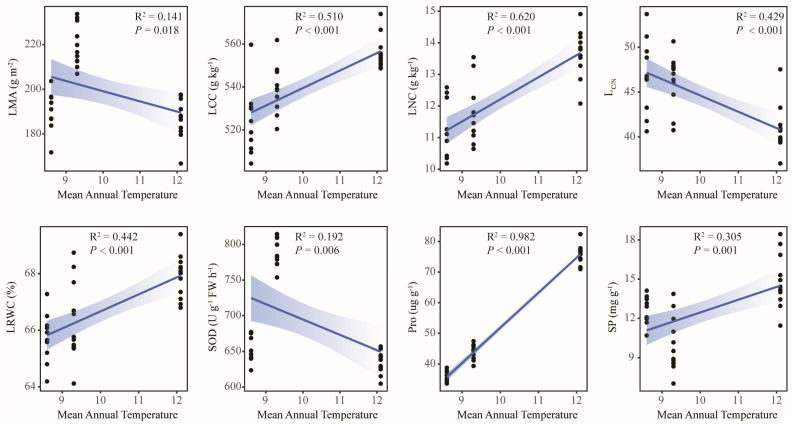
Linear regression analysis between leaf functional traits and mean annual temperature. LMA, leaf mass per area; LCC, contents of carbon; LNC, contents of nitrogen; L_C/N_, carbon nitrogen ratio; LRWC, leaf water content; SOD, superoxide dismutase activity; Pro, proline content; SP, soluble protein content.

This study conducted linear regression analyses to assess relationships between leaf functional traits and geographic/climatic factors. The results showed that most traits were significantly correlated with geographical and climatic factors, among which 16 traits, including MDA (R^2^ = 0.939, *p* < 0.001), POD (R^2^ = 0.753, *p* < 0.001), SOD (R^2^ = 0.733, *p* < 0.001), C_T_ (R^2^ = 0.698, *p* < 0.001), and LMA (R^2^ = 0.559, *p* < 0.001), were significantly correlated with Lat (Figure 5). A total of 17 traits, including POD (R^2^ = 0.744, *p* < 0.001), C_T_ (R^2^ = 0.580, *p* < 0.001), and LCC (R^2^ = 0.414, *p* < 0.001), were significantly correlated with ASL (Figure 6). A total of 19 traits, including POD (R^2^ = 0.888, *p* < 0.001), MDA (R^2^ = 0.848, *p* < 0.001), C_T_ (R^2^ = 0.791, *p* < 0.001), and SOD (R^2^ = 0.520, *p* < 0.001), were significantly correlated with MAP (Figure 7). In total, 8 traits, including Pro (R^2^ = 0.982, *p* < 0.001), LNC (R^2^ = 0.620, *p* < 0.001), and LCC (R^2^ = 0.510, *p* < 0.001), were significantly correlated with MAT (Figure 8). Notably, 10 traits (e.g., MDA, POD, and C_T_) responded consistently across all geographic parameters, while 4 traits (including SOD) showed universal sensitivity to climatic variables. Except for the above-mentioned traits, the sensitivity of the remaining traits to geographical and climatic factors was not significant (Figure S2–S5).

## 3. Discussion

### 3.1. Characteristics of Leaf Functional Traits of Old P. tabuliformis

Analysis of 28 functional traits of old *P. tabuliformis* revealed geographical heterogeneity in trait plasticity (Figure 1). Morphological and anatomical traits displayed low geographical variability, whereas almost all the chemical and physiological traits exhibited significant regional divergence (*p* < 0.05). Previous studies have shown that structural traits (morphological and anatomical traits) incur higher plasticity costs and minimal changes under environmental stress compared to reversible chemical and physiological traits [39]. In contrast, the “leaf economic spectrum” suggested that species with high leaf nitrogen content, high photosynthetic rate, fast respiration rate, and lower leaf specific weight were more inclined to the “fast investment-return” type [11]. In contrast, LNC, LPC, and C_T_ of old *P. tabuliformis* in Huanglong area remained at a high level, while LMA was relatively low, which may be more inclined to an acquisitive resource utilization strategy. This conclusion is consistent with the analysis results of the CSR strategy, the C-S axis in the CSR strategy reflects the trade-off between resource competition and stress tolerance of plants, which can correspond to the “fast-slow” economic pattern. The old trees in Huanglong exhibited a notably high C-strategy value (10.08%) and a correspondingly low S-strategy value (89.92%), indicating their strong resource acquisition capacity. More data are needed to verify this hypothesis in the future.

Plant functional traits exhibit interdependent coordination and trade-off relationships that optimize trait combinations for environmental adaptation [40]. In this study, morphological/anatomical traits and chemical/physiological traits diverged along two near-orthogonal directions in space, suggesting functional differentiation (Figure 2b). Morphological and anatomical traits predominantly govern resource acquisition strategies, reflecting the photosynthetic optimization-resource investment trade-off [41]. As LMA is a core component of the leaf economic spectrum, it signals resource-use efficiency, with higher LMA typically indicating more conservative resource-use strategies. In this study, the old trees in Shenmu exhibited significantly higher LMA values compared to those in Huanglong and Taibai regions. Given the harsh environmental conditions in Shenmu, plants are likely to adopt more conservative resource-use strategies to ensure survival, w. While vascular tissues (VBP, VBA) and RCN regulate photosynthetic product allocation [42]. Strong trait correlations (Figure 2a) indicate coordinated resource allocation patterns where morphological investments drive anatomical construction, subsequently directly or indirectly influencing photosynthesis—consistent with observations in *Picea asperata* Mast. Needles [43]. Notably, stomatal traits showed limited associations with morphological and anatomical traits in this study, mirroring findings by Liu et al. (2019) but contrasting reports from woody species in the Loess Plateau [44,45]. The absence of this trait relationship may be related to the multi-dimensional adaptation strategies of plants in unfavorable environments [46], with multiple directions of trait variation optimizing the availability of plants to limited resources. Chemical traits mediate mineral nutrient utilization and growth regulation [47], whereas physiological traits enable rapid stress responses [48]. The weak integration observed between chemical/physiological traits and other traits in this study (Figure 2a), exemplified by LKC, LRWC, and SS showing almost no significant correlations with other traits, may result from multidimensional adaptation and developmental constraints: while leaves exhibit plasticity during growth, morphological and anatomical traits become fixed post-maturation, unlike dynamically adjustable chemical and physiological traits. As a result, morphological and anatomical traits often fail to correlate closely with changes in chemical and physiological traits that occur on a daily or seasonal scale [49,50]. However, studies on leaf functional traits of *P. tabuliformis* plantation in the Loess Plateau showed that there was a significant positive correlation between physiological traits and anatomical traits [25]. This may be related to the unique adaptation mechanisms of old trees that promote stress resistance at the expense of growth [26].

In general, while leaf functional traits exhibit distinct roles in environmental adaptation, their synergistic interactions remain essential for plant survival. This study comprehensive multi-trait analysis provides critical insights into the trade-off mechanisms governing old tree acclimation to environmental stressors. However, given the multidimensional complexity of plant systems—encompassing trait networks, organ interactions, and developmental plasticity—exclusive focus on foliar traits (morphological, anatomical, chemical composition, and physiological parameters) limits holistic understanding of old trees in adapting to environmental changes. Future investigations should from a more comprehensive perspective to reveal old trees’ adaptation strategies.

### 3.2. Geo-Climate Model for Leaf Functional Traits

This study investigation of three geographically distinct sites revealed geo-climate models in leaf functional traits of old *P. tabuliformis*. From north to south, leaf morphological traits generally decreased gradually, and anatomical traits showed a trend of “high-low-high”. Contrastingly, chemical and physiological traits had no obvious uniform changes (Figure 1). This may be reflecting differential environmental sensitivity among traits. Physiological traits such as MDA, POD, and SOD were significantly correlated with Lat and MAP (R^2^ > 0.5, *p* < 0.001), and C_T_ and POD were also strongly influenced by ASL (R^2^ > 0.5, *p* < 0.001) (Figure 5, Figure 6 and Figure 7). This is supported by similar results reported in previous studies [51]. The observed Lat-ASL-MAP correlation (Table S2) highlights compounding climatic effects: both the decrease in Lat and the increase in ASL would affect the change in MAP. Elevated POD and SOD activities under low-MAP conditions suggest adaptive oxidative stress mitigation—consistent with xerophytic strategies [52]. Notably, C_T_ accumulation correlated positively with Lat, contrary to expected altitudinal suppression. The possible reasons for this pattern are as follows. First of all, C_T_ was affected by Lat and ASL changes; although the Taibai area was located in a low Lat area, ASL was significantly higher than that of the other two places. Previous studies have proved that ASL was significantly negatively correlated with C_T_ [53]. Secondly, C_T_ abundance and high interspecific variation in plants may also lead to changes in their geographical patterns [54]. In this study, substantial intraspecific C_T_ variation (CV = 31.69%) potentially skews regional means. Chemical traits including LNC and LCC demonstrated temperature sensitivity, correlating strongly with MAT (R^2^ > 0.5, *p* < 0.001). The positive effect of MAT on LCC is consistent with previous studies based on a global vascular plant dataset [55]. Although the response pattern of LNC to MAT is contrary to the results of previous studies in China [56], it is similar to the results in the alpine coniferous forest leaves on the Tibetan Plateau [57]. This pattern likely reflects adaptive resource allocation strategies in cold environments, where plants prioritize carbon investment in structural reinforcement to mitigate environment stressors. Furthermore, the pronounced spatial variability in the chemical traits of old trees may derive from heterogeneity interacting with stochastic environmental fluctuations. Morphological and anatomical traits showed limited climate correlations, constrained by species-specific developmental canalization. Although some morphological and anatomical traits have a certain correlation with geographical and climatic factors, the correlation is weak. However, even weak associations retain ecological relevance as supplemental environmental indicators. In addition, no correlation was observed between RCN and LKC with the measured geographical and climatic factors in this study. Research has demonstrated that genetic predisposition serves as the primary factor influencing RCN variability in trees [58]. While LKC exhibits associations with geographical and climatic conditions [59], this difference may be related to the size of the study area. And local-scale studies may lack the capacity to adequately capture variations in functional traits, thereby limiting their generalizability. Although the geo-climate models of leaf functional traits were discussed as comprehensively as possible in this study, their interpretation of changes in leaf functional traits of old trees is still limited, and plants need to be analyzed and discussed in the context of integrated environmental factors in the future.

### 3.3. The Main Driving Factors and Action Models of Leaf Functional Traits

Leaf functional trait variation typically arises from the joint regulation of phylogenetic and environmental drivers. Given the uncertain phylogenetic consistency among sampled old trees, we quantified phylogenetic signals across 28 traits using Pagel’s λ and Blomberg’s *K* statistic. All traits exhibited weak phylogenetic conservation (Table 2), demonstrating stronger environmental than evolutionary constraints during trait diversification [60]. Consequently, phylogenetic correction may be excluded when analyzing the driving factors of leaf functional traits.

The differential impacts of edaphic, geographical, and climatic factors on leaf functional traits were systematically investigated (Figure 3). RDA evaluated the independent effects of these factors (Figure 3a–c), identifying W, NH_4_^+^-H, and NO_3_^−^-N as the key drivers of leaf trait variability (*p* < 0.05). These findings align with prior studies in the Loess Plateau [61,62]. The Loess Plateau region has strong nitrogen and water limitations [63]. Water availability critically governs ecosystem processes [64], with multidimensional leaf trait shifts in arid regions reflecting adaptive water-use strategies [65]. Concurrently, nitrogen dynamics (NH_4_^+^-H, NO_3_^−^-N) modulate plant functional traits via metabolic pathways. Many studies have examined how changes in leaf traits after the addition of N are related to resource use [66,67]. Geographical (Lat, ASL) and climatic (MAP, MAT) factors further explained trait variation [28,68]. Latitudinal gradients indirectly shape traits through associated climatic and edaphic heterogeneity [53]. For example, at high latitudes, where temperatures are relatively low, precipitation is also relatively low—changes in temperature and precipitation are important factors affecting plant growth. Hierarchical partitioning analysis (Figure 3d) demonstrated stronger combined effects of environmental factors compared to isolated contributions. Synergistic interactions among edaphic, geographical, and climatic drivers predominantly governed leaf traits of old *P. tabuliformis*, contrasting assertions of soil-dominated control [31,69,70]. This may be due to the large climatic and geographical variations in the three study sites masking the independent effects of edaphic factors. Generally, the ability of climate factors to shape leaf traits is higher than that of edaphic factors on a large scale [71,72]. SEM corroborated this pattern (Figure 3e): edaphic factors exhibited no direct effects on principal components PC1/PC2, and only had a strong negative correlation with geographical factors. However, this does not mean that edaphic factors have no driving effect on leaf functional traits, and it still plays a role together with other factors. Climate and geography directly influenced PC2 (morphological and anatomical traits), consistent with limited plasticity in mature leaves. While multi-factor integration improved model fitness, substantial unexplained variance highlights unexplored drivers (e.g., anthropogenic disturbances, biotic interactions), necessitating expanded analytical frameworks in future studies.

This investigation demonstrated the leaf characteristics of old *P. tabuliformis* across the Loess Plateau region, and identified several traits, such as MDA, POD, and SOD, as sensitive indicators of geographical and climatic adaptation, and discussed the driving factors and influencing ways of the variation in leaf functional traits. In the future protection of old trees, it is possible to reshape the functional traits by changing environmental factors, such as regulating soil water, so as to guide shifts in their survival mechanisms and bolster resilience in senescing arbor populations. Future research should adopt a multiscale approach integrating the following: whole-plant response analysis (roots, stems, foliage), multidimensional trait evaluation (resource allocation, photosynthetic efficiency, phenological synchrony), and biotic-abiotic interaction modeling. Such a framework advances our understanding of the survival strategies of old trees in arid areas and promotes the scientific management and conservation of old trees.

## 4. Materials and Methods

### 4.1. Study Site and Experimental Design

This study was conducted in the Loess Plateau of China. Three latitudinally graded sampling sites were established, Taibai (TB), Huanglong (HL), and Shenmu (SM) (Figure S6). All sites had continental monsoon climate, and the soil types were mainly brown soil and wind-sand soil. According to the Köppen–Geiger standard, the climate types of Taibai, Huanglong, and Shenmu can be further classified into Cwa, Dwb, and BWk. Mean annual precipitation (MAP) ranges from 433.4 to 719.6 mm, and mean annual temperature (MAT) ranged from 8.6 to 12.1 °C—all data from the China Meteorological Data Service Center (https://data.cma.cn/, accessed on 1 January 2025). See Table 4 for more information.

In 2023 and 2024, all old *Pinus tabulaeformis* Carrière within the three sampling sites were investigated and counted. The old trees within the study area were analyzed using a health evaluation system [73]. Specifically, assessments were conducted across four key dimensions: trunk condition, branch and twig status, foliage status, and bole condition. Principal component analysis was then performed to determine indicator weights and calculate composite health scores. Subsequently, the healthier specimens were selected for further study, including 10 Taibai, 12 Huanglong, and 11 Shenmu. Both Taibai and Shenmu old trees occurred as isolated individuals. Although Huanglong old trees grow together, they maintain sufficient spacing and exhibit no canopy overlap. Only a small number of herbs (*Carex* spp.) grow around all sample trees. The details of the sample trees are shown in Table S3. At the growth peak (July), healthy and vigorous branches were selected from the cardinal directions (north, south, east, west) of the crown for each sample tree, and the needles that grew in the current year were collected, while soil samples from the surface (0–20 cm depth) were concurrently extracted along four directions centered on the trunk base [74]. To ensure representative sampling, samples collected from each tree’s four cardinal orientations were combined, yielding 33 composite samples (33 soil and 33 needle samples). The needles were subdivided into triplicate aliquots preserved under distinct storage regimes: 4 °C, −80 °C, and FAA fixed solution, for the determination of leaf functional traits and phylogenetic analysis. The soil samples were air-dried for soil physicochemical property analysis.

### 4.2. Analysis of Soil Physical and Chemical Properties

The physical and chemical properties of soil samples were determined using conventional methods [74]. Soil pH was determined using a glass electrode in 1:25 soil-aqueous solution. Fresh soil samples were subjected to drying in an oven maintained at 105 °C until a constant mass was achieved (12–14 h), and moisture content (W, %) was subsequently determined through a gravimetric approach based on mass reduction. Soil organic matter (SOC, g kg^−1^) was quantified by external heating with potassium dichromate. After digestion with H_2_SO_4_-H_2_O_2_, soil total nitrogen content (TN, g kg^−1^) and soil total phosphorus content (TP, g kg^−1^) were determined using AA3 continuous flow analyzer (AA3, SEAL Analytical, Norderstedt, Germany); soil total potassium content (TK, g kg^−1^) was determined using a flame photometer (Flame photometer, Sherwood, Cambridge, UK). Soil ammonium nitrogen (NH_4_^+^-N, mg kg^−1^) and nitrate nitrogen (NO_3_^−^-N, mg kg^−1^) were extracted using 1 M KCl and analyzed with the AA3 continuous flow analyzer. Soil available phosphorus (AP, mg kg^−1^) was extracted by 0.5 M Na_2_CO_3_ and determined using molybdenum blue colorimetry. Soil available potassium (AK, mg kg^−1^) was extracted with 1 M ammonium acetate solution and determined by flame spectrophotometry.

### 4.3. Determination of Leaf Functional Traits

All indicators and abbreviations used in the study are shown in Table 5.

Morphological traits: NL, NW, and NT were measured using digital display vernier calipers. NS and NV are calculated by treating a needle as half of a cylinder [75], and the formulas are (1) and (2). VSN was defined as the ratio of NV to NS, LMA was derived as the ratio of dry weight to leaf area.

(1) NS=1/2×π×NW×NL+NW×NL

(2) $$NV=1/2\times \pi \times {\left(NW/2\right)}^{2}\times NL$$

Anatomical traits: Needle segments (selecting the part 2 cm from the base) were processed using the nail polish impression method [76] to obtain epidermal imprints. Stomatal observations were conducted under a fluorescence microscope (Fluorescent biological microscope, COIC, Chongqing, China), and SD and SN on a 2 mm stomatal band were calculated (both concave and convex). The cross-sectional structure specimens of the needles were prepared by the freehand sectioning method and observed with a fluorescence microscope. A total of 6 key parameters were measured using I_MAGE_J software (v1.54i): RCN, VBA, NSP, NSA, VBP, and VBA. YQJB was the ratio of NSA to VBA.

Chemical traits: LCC was quantified by external heating with potassium dichromate; after digestion with H_2_SO_4_-H_2_O_2_, LNC and LPC were determined using AA3 continuous flow analyzer, LKC was determined using a flame photometer; L_C/N_ is the ratio of LCC to LNC [74].

Physiological traits: LRWC was assessed following the protocol of Gadallah [77]. C_T_ was determined via the 80% acetone extraction [78]. SOD activity was assayed using the nitro blue tetrazolium reduction [79]. POD activity was determined via guaiacol oxidation [80]. MDA was determined via thiobarbituric acid reaction [81]. Pro was analyzed via ninhydrin colorimetric [82]. SP was determined using the Coomassie Brilliant Blue G-250 [83]. SS was determined using the anthrone colorimetric [84].

### 4.4. Construction of Phylogenetic Tree

Leaf DNA was extracted using a DNA extraction kit (BioTeke, Beijing, China). Simple Sequence Repeat (SSR) analysis was conducted using 16 primers (Table S4), selected for robust amplification consistency and elevated polymorphism indices, as previously reported [85,86,87]. The PCR system was 25 μL, and the reaction procedure for PCR amplification was as follows: predenaturation at 98 °C for 2 min; denatured for 10 s at 98 °C; annealing at 50~60 °C for 15 s, delay at 72 °C for 20 s; 33 cycle; and finally, extension for 5 min at 72 °C and storage at 4 °C. Amplified DNA fragments underwent capillary electrophoretic separation for analytical characterization. Raw fluorescence signals were processed through GeneMarker v1.65 and genetic diversity parameters were calculated with GenAlex v6.502 [88,89]. Multivariate clustering utilizing Nei’s genetic distance matrices was conducted, while evolutionary relationships were reconstructed in MEGA v6.0 [90].

The 16 primer pairs used in this study generated an average of 3.83 alleles per pair. A total of 6 primer pairs exhibited medium polymorphism (0.25 ≤ PIC < 0.5), while 10 pairs showed high polymorphism (PIC ≥ 0.50). These results demonstrate that these markers can successfully characterize the genetic variation within old *P. tabuliformis* populations. Supplementary Materials (Table S5) provide comprehensive data on primers.

### 4.5. Quantification of the Competition-Stress-Tolerant-Weed Type (CSR) Strategy

Based on the measured leaf dry weight, leaf wet weight, and leaf area, the scores of competitive type (C)—stress-tolerant type (S)—weed type (R) of old *P. tabuliformis* in different regions were calculated using the “StrateFy” tool [91], and the key factors regulating the CSR strategy were analyzed through Mantel’s test.

### 4.6. Statistical Analysis

After preprocessing the data by the Normalization method ([0, 1]), the data was analyzed through the following methods. First, one-way analysis of variance (ANOVA) and the least significant difference (LSD) multiple comparison tests were conducted using IBM SPSS Statistics (v.26.0.0.0) software to analyze differences in leaf functional traits among old trees across distinct geographical regions. The “corrplot” and “FactoMineR” R packages were used to evaluate the correlations, synergies and trade-offs among leaf functional traits [92,93]. Secondly, phylogenetic signal analysis was conducted using the “phytools” R package to compute Blomberg’s *K* and Pagel’s λ [94]. Blomberg’s *K* values approximating 1 indicate trait evolution aligned with Brownian motion, suggesting a moderate phylogenetic signal or evolutionary conservation; Pagel’s λ = 1 implies trait variation is fully explained by phylogeny, whereas λ = 0 denotes no phylogenetic signal. Thirdly, the “vegan” R and “rdacca.hp” R packages were used for redundancy analysis (RDA) and hierarchical segmentation to explore the effects of edaphic, climatic and geographical factors on traits variation in different regions [95,96]. Direct and indirect effects of these factors were further analyzed using piecewise structural equation modeling (SEM). Finally, the “lm ()” function was used to explore the correlation between traits and environmental factors. All statistical procedures except ANOVA and LSD were performed in R version 4.0.2.

## 5. Conclusions

This study employed a geographical gradient approach to comparatively analyze 28 leaf functional traits in old *P. tabuliformis* across three distinct geographic regions of the Loess Plateau. The results demonstrate significant spatial heterogeneity in trait expression patterns, revealing that chemical and physiological traits exhibited greater environmental plasticity than structural traits. Multivariate analysis indicated that the integrated effects of environmental factors predominantly governed leaf functional trait variation, with geographical and climatic parameters exerting particularly pronounced direct effects on PC2 of trait variation. These results provide mechanistic insights into the adaptive strategies of long-lived conifers in water-limited ecosystems and contribute to predictive frameworks for vegetation dynamics under future climate scenarios. Furthermore, our findings offer empirical support for formulating science-based conservation strategies for ancient tree populations in arid regions.

## Figures and Tables

**Table 1:** Characteristics of leaf functional traits of old *P. tabuliformis*.

Category	Traits	Unit	Mean	SD	Min	Max	CV(%)
Morphological traits	NL	mm	96.16	17.26	65.05	127.70	17.95
	NW	mm	1.43	0.13	1.21	1.69	9.40
	NT	mm	0.86	0.07	0.75	1.00	8.17
	NS	mm^2^	357.15	91.57	203.28	551.91	25.64
	NV	mm^3^	79.63	27.51	37.75	141.76	34.55
	VSN	mm	0.22	0.02	0.18	0.26	9.40
	LMA	g m^−2^	198.65	17.33	166.78	233.67	8.72
Anatomical traits	SN	pcs	324.25	44.70	239.80	421.96	13.79
	SD	pcs mm^−2^	44.51	6.04	34.10	57.19	13.57
	RCN	pcs	9.84	1.22	7.80	13.77	12.35
	NSP	mm	3.81	0.32	3.27	4.69	8.39
	NSA	mm^2^	0.97	0.17	0.72	1.43	17.28
	VBP	mm	2.02	0.20	1.68	2.48	9.83
	VBA	mm^2^	0.29	0.06	0.20	0.43	20.14
	YQJB		3.36	0.27	2.91	4.09	8.09
Chemical traits	LCC	g kg^−1^	540.38	17.49	504.37	573.99	3.24
	LNC	g kg^−1^	12.28	1.37	10.18	14.91	11.14
	LPC	g kg^−1^	1.31	0.15	1.06	1.68	11.65
	LKC	g kg^−1^	7.59	1.07	5.53	9.64	14.02
	L_C/N_		44.42	4.23	37.05	53.69	9.52
Physiological traits	LRWC	%	66.73	1.38	64.12	69.40	2.07
	C_T_	mg g^−1^	0.52	0.16	0.24	0.80	31.69
	SOD	U g^−1^ FWh^−1^	692.32	71.85	604.81	814.49	10.38
	POD	u g^−1^ min^−1^	260.95	86.31	80.00	385.08	33.07
	MDA	umol g^−1^	17.55	4.71	9.19	23.80	26.85
	Pro	ug g^−1^	52.99	18.00	33.60	82.40	33.97
	SP	mg g^−1^	12.55	2.67	7.01	18.44	21.28
	SS	%	6.20	1.52	3.21	9.37	24.53

**Table 2:** Blomberg’s *K* and Pagel’s λ showing the phylogenetic signal of leaf functional traits.

Leaf Functional Traits		Blomberg’s *K*		Pagel’s λ	
		*K*	*p*-Value	λ	*p*-Value
Morphological traits	NL	0.79	0.84	0.00	1.00
	NW	0.79	0.83	0.00	1.00
	NT	0.80	1.00	0.00	1.00
	NS	0.78	1.00	0.00	1.00
	NV	0.78	1.00	0.00	1.00
	VSN	0.79	1.00	0.00	1.00
	LMA	0.89	0.67	0.00	1.00
Anatomical traits	SN	1.10	0.37	1.00	0.83
	SD	0.86	0.68	0.00	1.00
	RCN	1.21	0.16	1.00	0.56
	NSP	0.99	0.53	0.00	1.00
	NSA	0.96	0.68	0.00	1.00
	VBP	0.86	0.67	0.00	1.00
	VBA	0.85	1.00	0.00	1.00
	YQJB	0.89	0.64	0.00	1.00
Chemical traits	LCC	1.08	0.34	0.83	0.90
	LNC	1.20	0.33	1.00	0.57
	LPC	0.78	1.00	0.00	1.00
	LKC	0.88	0.66	0.00	1.00
	L_C/N_	1.22	0.32	1.00	0.55
Physiological traits	LRWC	1.18	0.14	1.00	0.61
	C_T_	0.78	1.00	0.00	1.00
	SOD	0.90	0.67	0.00	1.00
	POD	0.79	1.00	0.00	1.00
	MDA	0.79	1.00	0.00	1.00
	Pro	1.20	0.35	1.00	0.58
	SP	1.06	0.72	0.00	1.00
	SS	0.85	0.68	0.00	1.00

**Table 3:** Analysis of CSR strategy value of old *P. tabuliformis* in Different Regions.

Site Name	S (%)	C (%)	R (%)
SM	89.75 ^b^	10.25 ^a^	0.00
HL	89.92 ^b^	10.08 ^a^	0.00
TB	92.32 ^a^	7.68 ^b^	0.00

Different letters indicated significant differences in survival strategies among different populations (LSD test, *p* < 0.05).

**Table 4:** Geographical and climatic information of the study area.

Site Name	Lng	Lat	ASL (m)	MAP (mm)	MAT (°C)
SM	E 110°34′3″	N 38°27′9″	1098	433.4	9.3
HL	E 109°41′37″	N 35°38′26″	1084	561.4	12.1
TB	E 107°8′35″	N 34°6′24″	1508	719.6	8.6

Lng, Longitude; Lat, Latitude; ASL, Altitude.

**Table 5:** List of leaf functional traits included in the study with abbreviations and units.

Category	Traits	Abbreviation	Unit	Functional Significance
Morphological traits	needle length	NL	mm	The longitudinal and radial growth of needles.
	needle width	NW	mm	
	needle thickness	NT	mm	
	needle surface area	NS	mm^2^	Reflect the drought-resistant ability of plants.
	needle volume	NV	mm^3^	
	the ratio of NV to NS	VSN	mm	
	leaf mass per area	LMA	g m^−2^	Reflect plant tradeoff strategy.
Anatomical traits	stomata number	SN	pcs	Reflect the plant’s ability to breathe and transpiration.
	stomatal density	SD	pcs mm^−2^	
	resin canal number	RCN	pcs	Reflect the transport capacity of secondary metabolites.
	needle section perimeter	NSP	mm	Reflect the growth of water and nutrient-carrying tissue of coniferous leaves.
	needle section area	NSA	mm^2^	
	vascular bundle perimeter	VBP	mm	
	vascular bundle area	VBA	mm^2^	
	the ratio of NSA to VBA	YQJB		Photosynthesis and material transport capacity.
Chemical traits	contents of carbon	LCC	g kg^−1^	Comprehensive parameters to characterize plant growth and development.
	contents of nitrogen	LNC	g kg^−1^	
	contents of phosphorus	LPC	g kg^−1^	
	contents of potassium	LKC	g kg^−1^	
	carbon nitrogen ratio	L_C/N_		Reflect plant nutrient use efficiency.
Physiological traits	leaf water content	LRWC	%	Reflect the photosynthetic status of trees.
	total chlorophyll content	C_T_	mg g^−1^	
	superoxide dismutase activities	SOD	U g^−1^ FWh^−1^	Important protective enzymes in plants to remove reactive oxygen species.
	peroxidase activities	POD	u g^−1^ min^−1^	
	contents of malondialdehyde	MDA	umol g^−1^	Measuring the damage to the cell membrane of trees.
	contents of proline	Pro	ug g^−1^	Important osmoregulatory substances.
	soluble protein	SP	mg g^−1^	
	soluble sugar	SS	%	

## Data Availability

The original contributions presented in this study are included in the article. Further inquiries can be directed to the corresponding author.

## Supplementary Materials

The following supporting information can be downloaded at: https://www.mdpi.com/article/10.3390/plants14142128/s1, Table S1: Physical and chemical properties of soil in different regions; Table S2: Correlation analysis of geographical and climatic factors; Table S3: Plot information of old *P. tabuliformis*; Table S4: Sixteen primers and their sequence in the SSR-PCR of *P. tabuliformis*; Table S5: Parameters of genetic diversity for 16 SSR loci; Table S6: Parameters of genetic diversity for 3 regions of old *P. tabuliformis*; Figure S1: Genetic distance analysis of old *P. tabuliformis*. (a) Principal coordinate analysis for 3 regions of old *P. tabuliformis*. (b) UPGMA dendrogram of old *P. tabuliformis* germplasm resources; Figure S2: Linear regression analysis between leaf functional traits and latitude. NW, needle width; VSN, the ratio of NV to NS; SD, stomatal density; RCN, resin canal number; NSP, needle section perimeter; YQJB, the ratio of NSA to VBA; LCC, contents of carbon; LNC, contents of nitrogen; LKC, contents of potassium; L_C/N_, carbon nitrogen ratio; LRWC, leaf water content; Pro, proline content; Figure S3: Linear regression analysis between leaf functional traits and altitude. NW, needle width; VSN, the ratio of NV to NS; LMA, leaf mass per area; RCN, resin canal number; NSP, needle section perimeter; NSA, needle section area; VBP, vascular bundle perimeter; VBA, vascular bundle area; LKC, contents of potassium; SOD, superoxide dismutase activity; SP, soluble protein content; Figure S4: Linear regression analysis between leaf functional traits and mean annual precipitation. RCN, resin canal number; NSP, needle section perimeter; NSA, needle section area; YQJB, the ratio of NSA to VBA; LNC, contents of nitrogen; LKC, contents of potassium; L_C/N_, carbon nitrogen ratio; LRWC, leaf water content; Pro, proline content; Figure S5: Linear regression analysis between leaf functional traits and mean annual temperature. NL, needle length; NW, needle width; NT, needle thickness; NS, needle surface area; NV, needle volume; VSN, the ratio of NV to NS; SN, stomata number; SD, stomatal density; RCN, resin canal number; NSP, needle section perimeter; NSA, needle section area; VBP, vascular bundle perimeter; VBA, vascular bundle area; YQJB, the ratio of NSA to VBA; LPC, contents of phosphorus; LKC, contents of potassium; C_T_, total chlorophyll content; POD, peroxidase activity; MDA, malondialdehyde content; SS, soluble sugar content; Figure S6: Map of study area generated in ArcMap 10.3 (ESRI, Redlands, CA, USA); Figure S7: Study of the workflow diagram.

## References

[1] Piovesan G., Cannon C.H., Liu J., Munné-Bosch S. (2022). Ancient trees: Irreplaceable conservation resource for ecosystem restoration. Trends Ecol. Evol..

[2] Büntgen U., Krusic P.J., Piermattei A., Coomes D.A., Esper J., Myglan V.S., Kirdyanov A.V., Camarero J.J., Crivellaro A., Körner C. (2019). Limited capacity of tree growth to mitigate the global greenhouse effect under predicted warming. Nat. Commun..

[3] Camarero J.J., García-Ruiz J.M., Sangüesa-Barreda G., Galván J.D., Alla A.Q., Sanjuán Y., Beguería S., Gutiérrez E. (2015). Recent and Intense Dynamics in a Formerly Static Pyrenean Treeline. Arct. Antarct. Alp. Res..

[4] Lindenmayer D.B. (2017). Conserving large old trees as small natural features. Biol. Conserv..

[5] Cannon C.H., Piovesan G., Munné-Bosch S. (2022). Old and ancient trees are life history lottery winners and vital evolutionary resources for long-term adaptive capacity. Nat. Plants.

[6] Lindenmayer D.B., Laurance W.F., Franklin J.F. (2012). Global Decline in Large Old Trees. Science.

[7] McDowell N.G., Allen C.D., Anderson-Teixeira K., Aukema B.H., Bond-Lamberty B., Chini L., Clark J.S., Dietze M., Grossiord C., Hanbury-Brown A. (2020). Pervasive shifts in forest dynamics in a changing world. Science.

[8] Fu Q., Qiu E., Zhang Y., Huang L., Wang H., Jiang S. (2022). Discussion of the Distribution Pattern and Driving Factors of 2 Large Old Tree Resources in Beijing. Forests.

[9] Xie C., Jim C. (2025). Safeguarding China’s irreplaceable natural legacy: Combating the illicit trade of old trees. Environ. Conserv..

[10] Islam T., Hamid M., Nawchoo I.A., Khuroo A.A. (2024). Leaf functional traits vary among growth forms and vegetation zones in the Himalaya. Sci. Total Environ..

[11] Wright I.J., Reich P.B., Westoby M., Ackerly D.D., Baruch Z., Bongers F., Cavender-Bares J., Chapin T., Cornelissen J.H.C., Diemer M. (2004). The worldwide leaf economics spectrum. Nature.

[12] Puglielli G., Bricca A., Chelli S., Petruzzellis F., Acosta A.T.R., Bacaro G., Beccari E., Bernardo L., Bonari G., Bolpagni R. (2024). Intraspecific variability of leaf form and function across habitat types. Ecol. Lett..

[13] Funk J.L., Larson J.E., Ames G.M., Butterfield B.J., Cavender-Bares J., Firn J., Laughlin D.C., Sutton-Grier A.E., Williams L., Wright J. (2017). Revisiting the Holy Grail: Using plant functional traits to understand ecological processes. Biol. Rev..

[14] Wang Z., Zheng R., Yang L., Tan T., Li H., Liu M. (2022). Elevation gradient distribution of indices of tree population in a montane forest: The role of leaf traits and the environment. For. Ecosyst..

[15] Wang H., Wang R., Harrison S.P., Prentice I.C. (2022). Leaf morphological traits as adaptations to multiple climate gradients. J. Ecol..

[16] Santos K.R., Pereira M.P., Ferreira A.C.G., de Almeida Rodrigues L.C., de Castro E.M., Corrêa F.F., Pereira F.J. (2015). Typha domingensis Pers. growth responses to leaf anatomy and photosynthesis as influenced by phosphorus. Aquat. Bot..

[17] Pan S., Wang X., Yan Z., Wu J., Guo L., Peng Z., Wu Y., Li J., Wang B., Su Y. (2024). Leaf stomatal configuration and photosynthetic traits jointly affect leaf water use efficiency in forests along climate gradients. New Phytol..

[18] Liu X., Chen H., Sun T., Li D., Wang X., Mo W., Wang R., Zhang S. (2021). Variation in woody leaf anatomical traits along the altitudinal gradient in Taibai Mountain, China. Glob. Ecol. Conserv..

[19] Cai Q., Ji C., Yan Z., Jiang X., Fang J. (2017). Anatomical responses of leaf and stem of Arabidopsis thaliana to nitrogen and phosphorus addition. J. Plant Res..

[20] Lu J., Zhao X., Wang S., Feng S., Ning Z., Wang R., Chen X., Zhao H., Chen M. (2023). Untangling the influence of abiotic and biotic factors on leaf C, N, and P stoichiometry along a desert-grassland transition zone in northern China. Sci. Total Environ..

[21] Fan H., Wu J., Liu W., Yuan Y., Hu L., Cai Q. (2015). Linkages of plant and soil C:N:P stoichiometry and their relationships to forest growth in subtropical plantations. Plant Soil.

[22] Zhu D., Hui D., Wang M., Yang Q., Yu S. (2020). Light and competition alter leaf stoichiometry of introduced species and native mangrove species. Sci. Total Environ..

[23] Hu Y., Zuo X., Yue P., Zhao S., Guo X., Li X., Medina-Roldán E. (2020). Increased Precipitation Shapes Relationship between Biochemical and Functional Traits of Stipa glareosa in Grass-Dominated Rather than Shrub-Dominated Community in a Desert Steppe. Plants.

[24] Su Y., Cui B., Luo Y., Wang J., Wang X., Ouyang Z., Wang X. (2021). Leaf Functional Traits Vary in Urban Environments: Influences of Leaf Age, Land-Use Type, and Urban–Rural Gradient. Front. Ecol. Evol..

[25] Zhang Z., Wang X., Guo S., Li Z., He M., Zhang Y., Li G., Han X., Yang G. (2023). Divergent patterns and drivers of leaf functional traits of Robinia pseudoacacia and Pinus tabulaeformis plantations along a precipitation gradient in the Loess plateau, China. J. Environ. Manag..

[26] Pasques O., Munné-Bosch S. (2024). Ancient trees are essential elements for high-mountain forest conservation: Linking the longevity of trees to their ecological function. Proc. Natl. Acad. Sci. USA.

[27] Yan P., He N., Yu K., Xu L., Van Meerbeek K. (2023). Integrating multiple plant functional traits to predict ecosystem productivity. Commun. Biol..

[28] Chen X., Xie J., Wu Q., Zhang H., Li Y. (2024). Climate and soil explain contrasting intraspecific trait variability of widespread species over a large environmental gradient. Glob. Ecol. Conserv..

[29] Ren L., Huang Y., Pan Y., Xiang X., Huo J., Meng D., Wang Y., Yu C. (2022). Differential Investment Strategies in Leaf Economic Traits Across Climate Regions Worldwide. Front. Plant Sci..

[30] de la Riva E.G., Villar R., Pérez-Ramos I.M., Quero J.L., Matías L., Poorter L., Marañón T. (2018). Relationships between leaf mass per area and nutrient concentrations in 98 Mediterranean woody species are determined by phylogeny, habitat and leaf habit. Trees.

[31] Joswig J.S., Wirth C., Schuman M.C., Kattge J., Reu B., Wright I.J., Sippel S.D., Rüger N., Richter R., Schaepman M.E. (2022). Climatic and soil factors explain the two-dimensional spectrum of global plant trait variation. Nat. Ecol. Evol..

[32] Joshi R.K., Mishra A., Gupta R., Garkoti S.C. (2024). Leaf and tree age-related changes in leaf ecophysiological traits, nutrient, and adaptive strategies of Alnus nepalensis in the central Himalaya. J. Biosci..

[33] Ma Z., Guo D., Xu X., Lu M., Bardgett R.D., Eissenstat D.M., McCormack M.L., Hedin L.O. (2018). Evolutionary history resolves global organization of root functional traits. Nature.

[34] Gao X., Dai J., Shahzad K., Wang H., Tao Z., Alatalo J.M. (2022). Association of spring phenological traits with phylogeny and adaptation to native climate in temperate plant species in Northeast China. Ecol. Indic..

[35] An N., Lu N., Fu B., Wang M., He N. (2021). Distinct Responses of Leaf Traits to Environment and Phylogeny Between Herbaceous and Woody Angiosperm Species in China. Front. Plant Sci..

[36] Wu Y., Liu L., Yin M., Guo W. (2022). Phylogenetic relationship and soil salinity shape intraspecific trait variability of Phragmites australis in the Yellow River Delta. Front. Mar. Sci..

[37] Sun J., Li G., Zhang Y., Qin W., Wang M. (2022). Identification of priority areas for afforestation in the Loess Plateau region of China. Ecol. Indic..

[38] Su J., Xiao S., Peng X., Che C., Zhao P. (2024). Responses of radial growth to climate change for two dominant artificial coniferous trees. Dendrochronologia.

[39] Stotz G.C., Salgado-Luarte C., Escobedo V.M., Valladares F., Gianoli E. (2021). Global trends in phenotypic plasticity of plants. Ecol. Lett..

[40] Yang Y., Kang L., Zhao J., Qi N., Li R., Wen Z., Kassout J., Peng C., Lin G., Zheng H. (2021). Quantifying Leaf Trait Covariations and Their Relationships with Plant Adaptation Strategies along an Aridity Gradient. Biology.

[41] Tian M., Yu G., He N., Hou J. (2016). Leaf morphological and anatomical traits from tropical to temperate coniferous forests: Mechanisms and influencing factors. Sci. Rep..

[42] Sack L., Scoffoni C., John G.P., Poorter H., Mason C.M., Mendez-Alonzo R., Donovan L.A. (2013). How do leaf veins influence the worldwide leaf economic spectrum? Review and synthesis. J. Exp. Bot..

[43] Wang J., Ma J., OuYang F., Wang J., Song L., Kong L., Zhang H. (2021). Instrinsic relationship among needle morphology, anatomy, gas exchanges and tree growth across 17 Picea species. New For..

[44] Liu C., Li Y., Xu L., Chen Z., He N. (2019). Variation in leaf morphological, stomatal, and anatomical traits and their relationships in temperate and subtropical forests. Sci. Rep..

[45] Yin Q., Wang L., Lei M., Dang H., Quan J., Tian T., Chai Y., Yue M. (2018). The relationships between leaf economics and hydraulic traits of woody plants depend on water availability. Sci. Total Environ..

[46] Liu X., Wang X., Zhu J., Wang X., Chen K., Yuan Y., Yang X., Mo W., Wang R., Zhang S. (2024). Strong conservatism in leaf anatomical traits and their multidimensional relationships with leaf economic traits in grasslands under different stressful environments. Ecol. Process..

[47] Güsewell S. (2004). N: P ratios in terrestrial plants: Variation and functional significance. New Phytol..

[48] Wang S., Zhou H., He Z., Ma D., Sun W., Xu X., Tian Q. (2024). Effects of Drought Stress on Leaf Functional Traits and Biomass Characteristics of Atriplex canescens. Plants.

[49] Paine C.E.T., Amissah L., Auge H., Baraloto C., Baruffol M., Bourland N., Bruelheide H., Daïnou K., de Gouvenain R.C., Doucet J.-L. (2015). Globally, functional traits are weak predictors of juvenile tree growth, and we do not know why. J. Ecol..

[50] Fortunel C., Stahl C., Heuret P., Nicolini E., Baraloto C. (2020). Disentangling the effects of environment and ontogeny on tree functional dimensions for congeneric species in tropical forests. New Phytol..

[51] Dong X., Shi L., Bao S., Ren Y., Fu H., You Y., Li Q., Chen Z. (2024). Comprehensive evaluation of freezing tolerance in prickly ash and its correlation with ecological and geographical origin factors. Sci. Rep..

[52] Huang W.-D., He Y.-Z., Wang H.-H., Zhu Y.-Z. (2022). Leaf Physiological Responses of Three Psammophytes to Combined Effects of Warming and Precipitation Reduction in Horqin Sandy Land, Northeast China. Front. Plant Sci..

[53] Ling-Ling S., Qing T., Guang L., Zong-Xing L., Xiaoying L., Juan G., Yuchen L., Qiao C., Yue Z. (2022). Variation in characteristics of leaf functional traits of alpine vegetation in the Three-River Headwaters Region, China. Ecol. Indic..

[54] Li Y., He N., Hou J., Xu L., Liu C., Zhang J., Wang Q., Zhang X., Wu X. (2018). Factors Influencing Leaf Chlorophyll Content in Natural Forests at the Biome Scale. Front. Ecol. Evol..

[55] Ma S., He F., Tian D., Zou D., Yan Z., Yang Y., Zhou T., Huang K., Shen H., Fang J. (2018). Variations and determinants of carbon content in plants: A global synthesis. Biogeosciences.

[56] Han W., Fang J., Guo D., Zhang Y. (2005). Leaf nitrogen and phosphorus stoichiometry across 753 terrestrial plant species in China. New Phytol..

[57] Ding J., Wang Q., Ge W., Liu Q., Kong D., Yin H. (2024). Coordination of leaf and root economic space in alpine coniferous forests on the Tibetan Plateau. Plant Soil.

[58] Rosner S., Hannrup B. (2004). Resin canal traits relevant for constitutive resistance of Norway spruce against bark beetles: Environmental and genetic variability. For. Ecol. Manag..

[59] Wang Z., Chen Z., Wu B., Liang Y., Wang G., Lin X., Yang J., Cheng Q., Wang J. (2024). Leaf stoichiometry of potassium, calcium, and magnesium in tropical plants: Responses to climatic and geographical variations—A case study from Hainan Island. Land Degrad. Dev..

[60] Blomberg S.P., Garland T., Ives A.R. (2003). Testing for Phylogenetic Signal in Comparative Data: Behavioral Traits are More Labile. Evolution.

[61] Chai Y., Dang H., Yue M., Xu J., Zhang L., Quan J., Guo Y., Li T., Wang L., Wang M. (2019). The role of intraspecific trait variability and soil properties in community assembly during forest secondary succession. Ecosphere.

[62] Zhang Q., Wei W., Chen L., Yang L., Chen H.Y.H., Luo Y. (2020). Soil Water Availability Drives Changes in Community Traits Along a Hydrothermal Gradient in Loess Plateau Grasslands. Rangel. Ecol. Manag..

[63] Wang J., Wen X. (2022). Limiting resource and leaf functional traits jointly determine distribution patterns of leaf intrinsic water use efficiency along aridity gradients. Front. Plant Sci..

[64] Deng L., Wang K., Li J., Zhao G., Shangguan Z. (2016). Effect of soil moisture and atmospheric humidity on both plant productivity and diversity of native grasslands across the Loess Plateau, China. Ecol. Eng..

[65] Wang J., Wen X., Lyu S., Guo Q. (2021). Transitioninmulti-dimensionalleaftraitsandtheircontrolsonwaterusestrategiesofco-occurringspecies along asoillimiting-resourcegradient. Ecol. Indic..

[66] Zhao C., Lin Q., Tian D., Ji C., Shen H., Fan D., Wang X., Fang J. (2023). Nitrogen addition promotes conservative resource-use strategies via aggravating phosphorus limitation of evergreen trees in subtropical forest. Sci. Total Environ..

[67] Zou Y., Li B., Peñuelas J., Sardans J., Yu H., Chen X., Deng X., Cheng D., Zhong Q. (2022). Response of functional traits in Machilus pauhoi to nitrogen addition is influenced by differences of provenances. For. Ecol. Manag..

[68] Zhou X., Xin J., Huang X., Li H., Li F., Song W. (2022). Linking Leaf Functional Traits with Soil and Climate Factors in Forest Ecosystems in China. Plants.

[69] Shao S., Li G., Wang J., Wang Y., Qu M., Zhao H., Zhu W., Li J. (2024). Temperature and soil attributes drive the regional variation in leaf anatomical traits of Populus euphratica. Glob. Ecol. Conserv..

[70] Xie G., Wang Y., Chen Z., Ji Y., Lu Y., Liang Y., Zhou R., Tao J. (2025). Response of Plant Leaf Traits to Environmental Factors in Climax Communities at Varying Latitudes in Karst Regions. Plants.

[71] Chelli S., Ottaviani G., Simonetti E., Wellstein C., Canullo R., Carnicelli S., Andreetta A., Puletti N., Bartha S., Cervellini M. (2019). Climate is the main driver of clonal and bud bank traits in Italian forest understories. Perspect. Plant Ecol. Evol. Syst..

[72] Gong H., Cui Q., Gao J. (2020). Latitudinal, soil and climate effects on key leaf traits in northeastern China. Glob. Ecol. Conserv..

[73] Zhao Z. (2021). Theory and Technology of Ancient Tree Protection.

[74] Bao S.D. (2000). Soil Agrochemical Analysis.

[75] Shi Y., Yu X., Wang X., Zhang J. (2013). The effects of stand structure on specific needle area in closed-canopy Chinese pine plantations. J. For. Res..

[76] Lai M., Dong L., Su R., Zhang L., Jia T., Chen T., Yi M. (2023). Needle functional features in contrasting yield phenotypes of slash pine at three locations in southern China. Ind. Crops Prod..

[77] Gadallah M.A.A. (1995). Effect of water stress, abscisic acid and proline on cotton plants. J. Arid. Environ..

[78] Arnon D.I. (1949). Copper Enzymes in Isolated Chloroplasts. Polyphenoloxidase in Beta Vulgaris. Plant Physiol..

[79] Giannopolitis C.N., Ries S.K. (1977). Superoxide Dismutases: II. Purification and Quantitative Relationship with Water-soluble Protein in Seedlings 1 2. Plant Physiol..

[80] Hammerschmidt R., Kuć J. (1982). Lignification as a mechanism for induced systemic resistance in cucumber. Physiol. Plant Pathol..

[81] Dipierro S., De Leonardis S. (1997). The ascorbate system and lipid peroxidation in stored potato (Solanum tuberosum L.) tubers. J. Exp. Bot..

[82] Bates L.S., Waldren R.P., Teare I.D. (1973). Rapid determination of free proline for water-stress studies. Plant Soil.

[83] Snyder J.C., Desborough S.L. (1978). Rapid estimation of potato tuber total protein content with coomassie brilliant blue G-250. Theor. Appl. Genet..

[84] Deng S.P., Tabatabai M.A. (1994). Colorimetric determination of reducing sugars in soils. Soil Biol. Biochem..

[85] Wu W., He K., Di H., Niu S., Ma Y., Zhang Z., Li Y. (2018). Genetic structure and geographic system of geographical population of *Pinus tabuliformis* mountain range based on SSR in Shanxi Province of northern China. J. Beijing For. Univ..

[86] Wang J., Guo S., Zhang Y., Zhang F., Yun Y., Zhang G. (2023). Transcriptome Analysis and Novel EST-SSR Marker Development for Pinus tabuliformis Seedlings from Four Provenances. Forests.

[87] Yang B., Niu S., El-Kassaby Y.A., Li W. (2021). Monitoring genetic diversity across Pinus tabuliformis seed orchard generations using SSR markers. Can. J. For. Res..

[88] Hulce D., Li X., Snyder-Leiby T., Johathan Liu C.S. (2011). GeneMarker® Genotyping Software: Tools to Increase the Statistical Power of DNA Fragment Analysis. J. Biomol. Tech..

[89] Peakall R., Smouse P.E. (2012). Genalex 6.5: Genetic analysis in Excel. Population genetic software for teaching and research-an update. Bioinformatics.

[90] Tamura K., Stecher G., Peterson D., Filipski A., Kumar S. (2013). MEGA6: Molecular Evolutionary Genetics Analysis Version 6.0. Mol. Biol. Evol..

[91] Pierce S., Negreiros D., Cerabolini B.E.L., Kattge J., Díaz S., Kleyer M., Shipley B., Wright S.J., Soudzilovskaia N.A., Onipchenko V.G. (2017). A global method for calculating plant CSR ecological strategies applied across biomes world-wide. Funct. Ecol..

[92] Wei T., Simko V. (2017). R Package “Corrplot”: Visualization of a Correlation Matrix. https://github.com/taiyun/corrplot.

[93] Lê S., Josse J., Husson F. (2008). FactoMineR: An R Package for Multivariate Analysis. J. Stat. Softw..

[94] Revell L.J. (2012). phytools: An R package for phylogenetic comparative biology (and other things). Methods Ecol. Evol..

[95] Oksanen J., Kindt R., Legendre P., O’Hara R. (2006). Vegan: Community Ecology Package, Version 1.8-1. https://CRAN.R-project.org/package=vegan.

[96] Lai J., Zou Y., Zhang J., Peres-Neto P. (2022). Generalizing hierarchical and variation partitioning in multiple regression and canonical analyses using the rdacca.hp R package. Methods Ecol. Evol..

