# Supplementary material for: Climatic Adaptability Changes in Leaf Functional Traits of Old Pinus tabulaeformis in Loess Plateau
Source: Plants (Basel). 2025 Jul 10;14(14):2128. doi: 10.3390/plants14142128 (PMC12300061; doi:10.3390/plants14142128)
Supplement: Supplementary file 1 [file plants-14-02128-s001.zip › plants-3716832-supplementary.pdf]

Table S1 Physical and chemical properties of soil in different regions.

Site name	W (%)	SOC (g kg^{-1})	TN (g kg^{-1})	TP (g kg^{-1})	TK (g kg^{-1})	AP (mg kg^{-1})	AK (mg kg^{-1})	NH_4^+-N (mg kg^{-1})	NO_3^--N (mg kg^{-1})	PH	C/N
SM	4.39±0.9^{c}	9.96±2.67^{b}	0.56±0.29^{b}	0.53±0.05^{b}	6.23±0.44^{c}	2.92±1.26	146.40±11.23^{c}	9.06±1.10^{b}	6.73±0.56^{b}	8.52±0.08^{a}	11.79±3.69
HL	11.14±0.93^{b}	16.95±2.38^{a}	1.03±0.23^{a}	0.61±0.02^{ab}	8.05±0.62^{b}	3.79±1.27	184.62±9.68^{b}	10.52±1.07^{a}	10.11±0.59^{a}	8.37±0.05^{b}	9.94±1.99
TB	13.99±1.25^{a}	17.53±3.19^{a}	1.17±0.50^{a}	0.72±0.23^{a}	12.66±1.14^{a}	3.33±0.85	213.58±10.15^{a}	6.04±1.88^{c}	6.40±1.16^{b}	8.24±0.18^{c}	9.71±2.99

Values are mean ± SD. Different letters indicated significant differences in leaf traits among different populations (LSD test, $P < 0.05$).

Table S2 Correlation analysis of geographical and climatic factors.

	Lat	ASL	MAP	MAT
Lat		-0.68**	-0.97**	-0.02
ASL	1.51E-05		0.82**	-0.64**
MAP	1.31E-20	4.31E-09		-0.22
MAT	0.93	7.12E-05	0.23	

***$P < 0.001$; **$P < 0.01$; *$P < 0.05$.

Table S3 Plot information of old *P. tabuliformis*.

Region	Sample	Lng	Lat	H (m)	DBH (cm)	P (m)	Age
SM	SG1	E 110°34'3"	N 38°27'9"	13.3	51.1	14.7	167
	SG2	E 110°34'3"	N 38°27'9"	8.9	77.4	13.1	162
	SG3	E 110°38'29'	N 38°27'26"	10.8	47.5	11.6	116
	SG4	E 110°38'40"	N 38°27'37"	11.7	53.0	9.0	145
	SG5	E 110°38'52"	N 38°28'11"	12.5	55.9	10.8	140
	SG6	E 110°42'35"	N 38°27'2"	11.0	46.2	10.6	154
	SG7	E 110°44'50"	N 38°28'30"	12.5	37.2	8.3	151
	SG8	E 110°27'49"	N 38°53'53"	11.8	97.1	13.0	358
	SG9	E 110°27'49"	N 38°53'53"	14.2	78.6	11.9	303
	SG10	E 110°34'33"	N 38°57'57"	12.3	109.2	20.5	252
	SG11	E 110°37'2"	N 38°58'39"	13.4	95.8	16.2	204
HL	HG1	E 109°41′37″	N 35°38′26″	16.0	80.3	13.7	131
	HG2	E 109°41′37″	N 35°38′26″	11.2	125.7	14.0	169
	HG3	E 109°41′37″	N 35°38′26″	13.1	90.5	13.5	195
	HG4	E 109°41′37″	N 35°38′26″	9.6	70.9	9.2	214
	HG5	E 109°41′37″	N 35°38′26″	14.4	77.6	9.3	209
	HG6	E 109°41′37″	N 35°38′26″	13.6	93.5	12.9	167
	HG7	E 109°41′37″	N 35°38′26″	12.8	73.1	10.9	206
	HG8	E 109°41′37″	N 35°38′26″	14.4	95.0	12.6	213
	HG9	E 109°41′37″	N 35°38′26″	13.6	64.0	12.9	199
	HG10	E 109°41′37″	N 35°38′26″	11.5	60.3	11.3	138
	HG11	E 109°41′37″	N 35°38′26″	12.8	47.3	11.1	127
	HG12	E 109°41′37″	N 35°38′26″	13.6	70.3	15.3	147
TB	TG1	E 107°8′35″	N 34°6′24″	7.8	98.7	16.8	210
	TG2	E 107°7′24″	N 34°1′35″	11.4	54.1	15.0	137
	TG3	E 107°7′25″	N 33°59′51″	15.0	94.0	16.8	190
	TG4	E 107°7′30″	N 33°59′54″	13.0	49.2	11.0	151
	TG5	E 107°7′32″	N 33°59′58″	12.7	124.3	13.5	234
	TG6	E 107°7′47″	N 33°59′0″	7.8	56.1	9.5	153
	TG7	E 107°7′35″	N 34°0′1″	7.8	79.6	11.0	203
	TG8	E 107°7′8″	N 33°59′17″	14.7	65.9	16.2	163
	TG9	E 107°10′7″	N 33°53′33″	12.3	48.4	10.5	116
	TG10	E 107°10′16″	N 33°51′42″	10.3	55.7	10.2	142

H, Height; DBH, Diameter at breast height; P, Crown breadth.

Table S4 16 primers and their sequence in the SSR-PCR of *P. tabuliformis*.

Primer	Primer sequence（5'→3'）		Fluorescent
g6405	F:-TCGGTGATGGTACCTGAACC	R:-GCCTATCAACGGCATCTTCG	FAM (Blue)
g6422	F:-TAATGCCGAGGAGGAGGAGG	R:-TTGGTAGGCCTATCAACGGC	FAM (Blue)
g7466	F:-GGGTGATGGACAGGCTCTTC	R:-CCCGAGTTCGAATTCTCCGA	FAM (Blue)
g10617	F:-TGACAGATGATCAAGGGGCG	R:-GAGCTGTGGCCTCAAGTTCT	FAM (Blue)
gp01	F:-ACAGATTTCATTCGGATTGCTT	R:-TCCATTGCTCTGATAAGCATGT	FAM (Blue)
pita_est0310	F:-AGGAGGGGTAAAGGCGAGA	R:-TGGAAGGGCACCAAGTTC	FAM (Blue)
pita_est0439	F:-TTGAACTAAAGGTAGAAAGAAAGA	R:-CATGGGAAACAGTAGCAGG	FAM (Blue)
pt0119	F:-AGAGAGGGTGATGGACAGG	R:-CGAGCTTAAGTTCGGAGAC	FAM (Blue)
pt0324	F:-AGATTTATATCTCTGAACTCCATG	R:-CTCCTCCGTCTGCCTTGC	FAM (Blue)
pt0448	F:-GGTAGATGAAATCGAAAAAACC	R:-CAAAGACGGAGACAAGAAGAA	FAM (Blue)
J9	F:-GTTTGCAGTGAAAGCATGAAAG	R:-GCACCAATTCCTTCTCAAATTC	FAM (Blue)
J12	F:-TATGCATGTAACGGTAGCCTTG	R:-GCAATTGTTCTATGGTCAGGGT	ROX(Red)
J20	F:-CACCTCCGTAGTTTGATGTTCC	R:-CGATGTATCGTGTACACAGCCT	FAM (Blue)
J42	F:-AACCTGTCATCCAGTTCCTGTT	R:-TTGTCAAATTCCAATTCAGCAC	TAMRA (Black)
J48	F:-GAAGAGGAAGACGAAATGGATG	R:-CTTTACATTTACCGCCTCTGCT	ROX(Red)
J50	F:-TCATCCATTTCAATAGCACGAC	R:-GTAGCTGCTTGGCCTGATTATC	HEX (Green)

Table S5 Parameters of genetic diversity for 16 SSR loci

SSR Loci	*Na*	*Ne*	*I*	*Ho*	*He*	*PIC*
g6405	6.00	3.68	1.51	0.70	0.73	0.79
g6422	6.00	3.84	1.52	0.29	0.73	0.84
g7466	4.67	2.89	1.21	0.45	0.62	0.65
g10617	3.00	2.66	1.01	0.70	0.61	0.61
gp01	6.00	3.53	1.47	1.00	0.72	0.69
pita_est0310	3.00	2.33	0.95	0.43	0.57	0.51
pita_est0439	4.33	2.62	1.12	0.47	0.59	0.61
pt0119	4.33	3.05	1.20	0.56	0.63	0.64
pt0324	6.00	4.46	1.62	0.76	0.77	0.76
pt0448	4.33	2.21	1.01	0.53	0.54	0.56
J9	2.00	1.42	0.43	0.35	0.27	0.25
J12	3.00	1.45	0.58	0.31	0.31	0.30
J20	2.00	1.53	0.46	0.16	0.28	0.27
J42	2.00	1.47	0.44	0.06	0.29	0.28
J48	2.67	2.11	0.81	0.78	0.51	0.46
J50	2.00	1.62	0.54	0.41	0.36	0.32
Mean	3.83	2.56	0.99	0.50	0.53	0.53

N_a, observed number of alleles; N_e, effective number of alleles; *I*, Shannon's information index; H_o, observed heterozygosity; H_e, expected heterozygosity; PIC, polymorphism information content.

Table S6 Parameters of genetic diversity for 3 regions of old *P. tabuliformis*.

Populations	*Na*	*Ne*	*I*	*Ho*	*He*	*Fis*	*F*
SM	3.75	2.62	1.01	0.48	0.55	0.13	0.13
HL	3.56	2.27	0.91	0.47	0.50	0.05	0.07
TB	4.19	2.77	1.05	0.54	0.55	0.01	-0.01
Mean	3.83	2.56	0.99	0.50	0.53	0.07	0.06

N_a, observed number of alleles; N_e, effective number of alleles; *I*, Shannon's information index; H_o, observed heterozygosity; H_e, expected heterozygosity; F_{is}, inbreeding coefficient at the population level; *F*, Fixation Index.

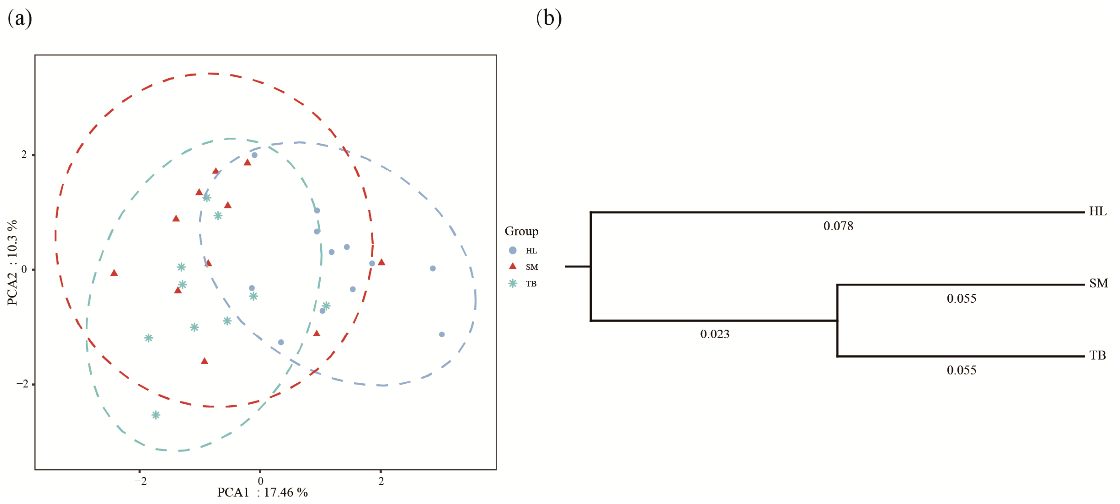

Figure S1 Genetic distance analysis of old *P. tabuliformis*. (a) Principal coordinate analysis for 3 regions of old *P. tabuliformis*. (b) UPGMA dendrogram of old *P. tabuliformis* germplasm resources.

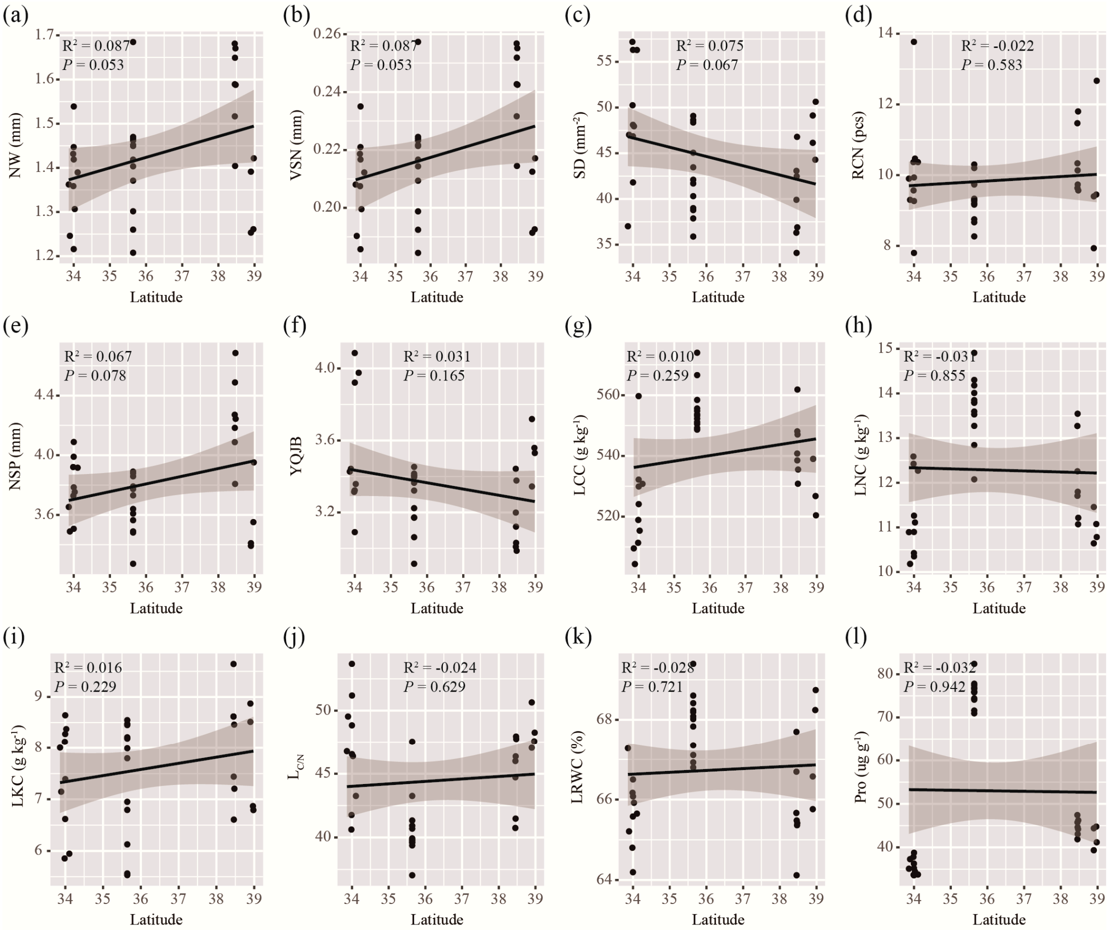

Figure S2 Linear regression analysis between leaf functional traits and latitude. NW, needle width; VSN, the ratio of NV to NS; SD, stomatal density; RCN, resin canal number; NSP, needle section perimeter; YQJB, the ratio of NSA to VBA; LCC, contents of carbon; LNC, contents of nitrogen; LKC, contents of potassium; $L_{C/N}$, carbon nitrogen ratio; LRWC, leaf water content; Pro, proline content.

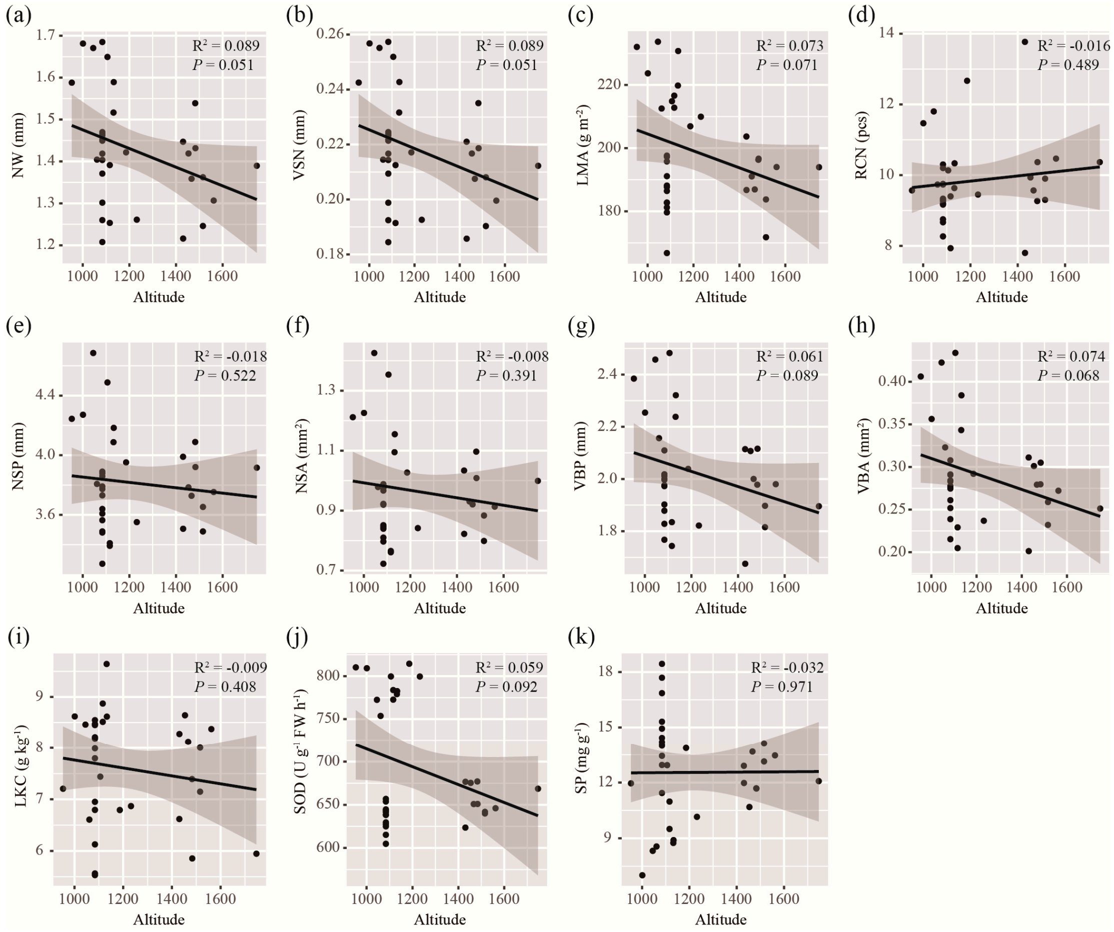

Figure S3 Linear regression analysis between leaf functional traits and altitude. NW, needle width; VSN, the ratio of NV to NS; LMA, leaf mass per area; RCN, resin canal number; NSP, needle section perimeter; NSA, needle section area; VBP, vascular bundle perimeter; VBA, vascular bundle area; LKC, contents of potassium; SOD, superoxide dismutase activity; SP, soluble protein content.

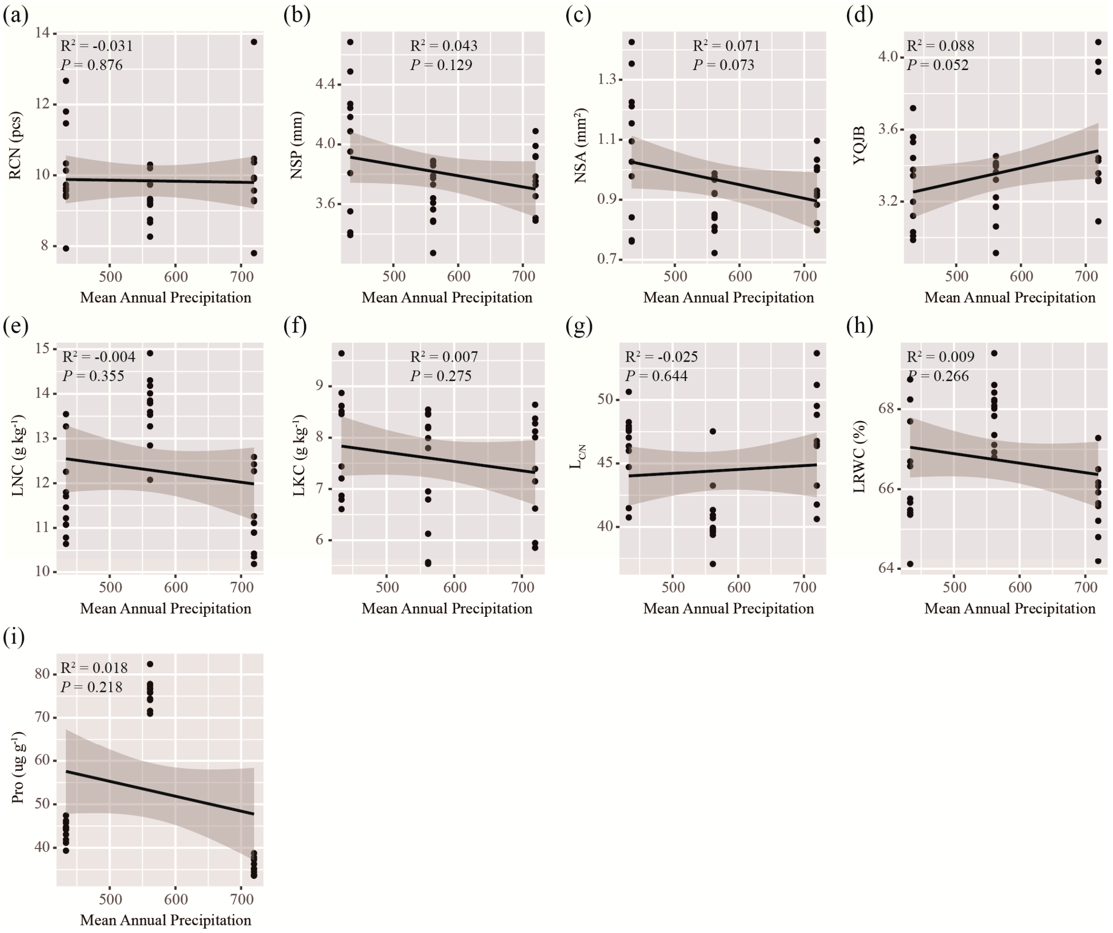

Figure S4 Linear regression analysis between leaf functional traits and mean annual precipitation. RCN, resin canal number; NSP, needle section perimeter; NSA, needle section area; YQJB, the ratio of NSA to VBA; LNC, contents of nitrogen; LKC, contents of potassium; $L_{C/N}$, carbon nitrogen ratio; LRWC, leaf water content; Pro, proline content.

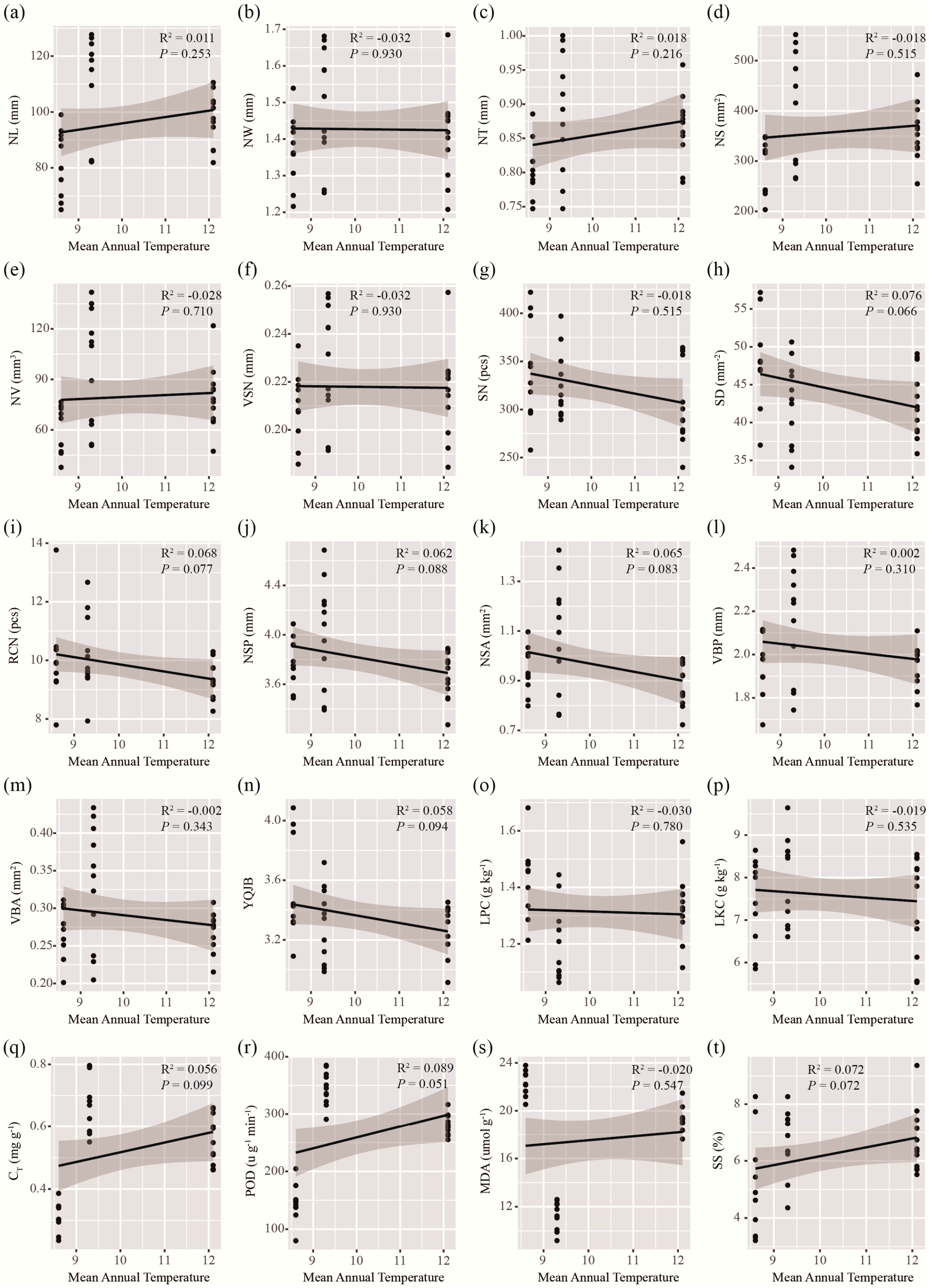

Figure S5 Linear regression analysis between leaf functional traits and mean annual temperature. NL, needle length; NW, needle width; NT, needle thickness; NS, needle surface area; NV, needle volume; VSN, the ratio of NV to NS; SN, stomata number; SD, stomatal density; RCN, resin canal number; NSP, needle section perimeter; NSA,

needle section area; VBP, vascular bundle perimeter; VBA, vascular bundle area; YQJB, the ratio of NSA to VBA; LPC, contents of phosphorus; LKC, contents of potassium; C_T, total chlorophyll content; POD, peroxidase activity; MDA, malondialdehyde content; SS, soluble sugar content.

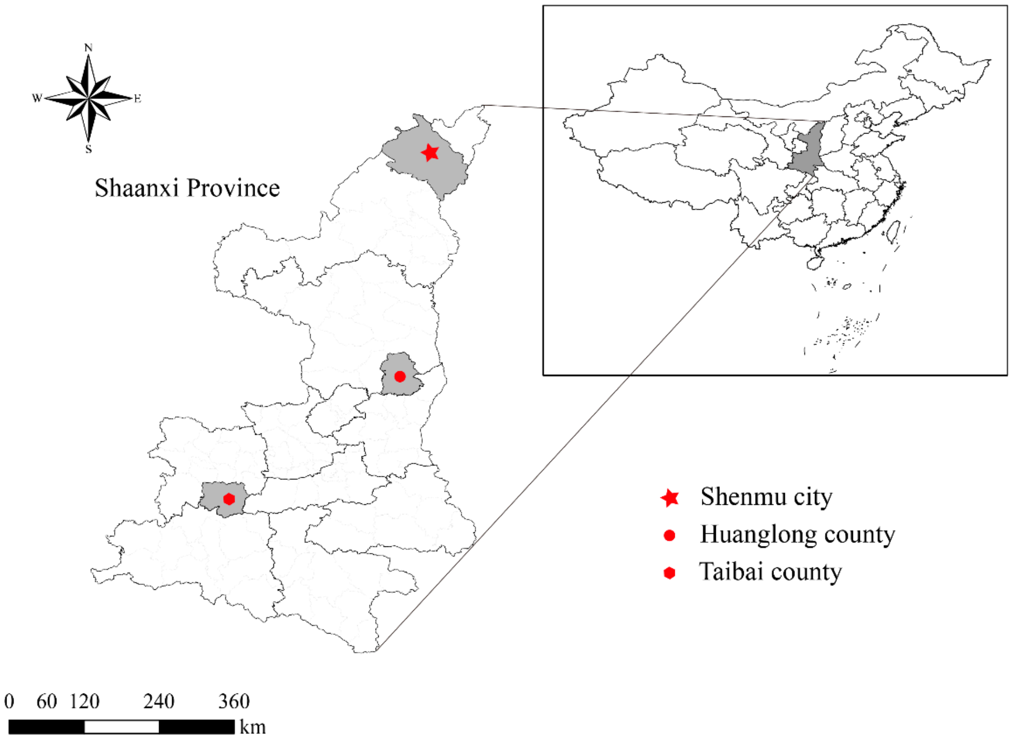

Figure S6 Map of study area generated in ArcMap 10.3 (ESRI, Redlands, CA, USA).

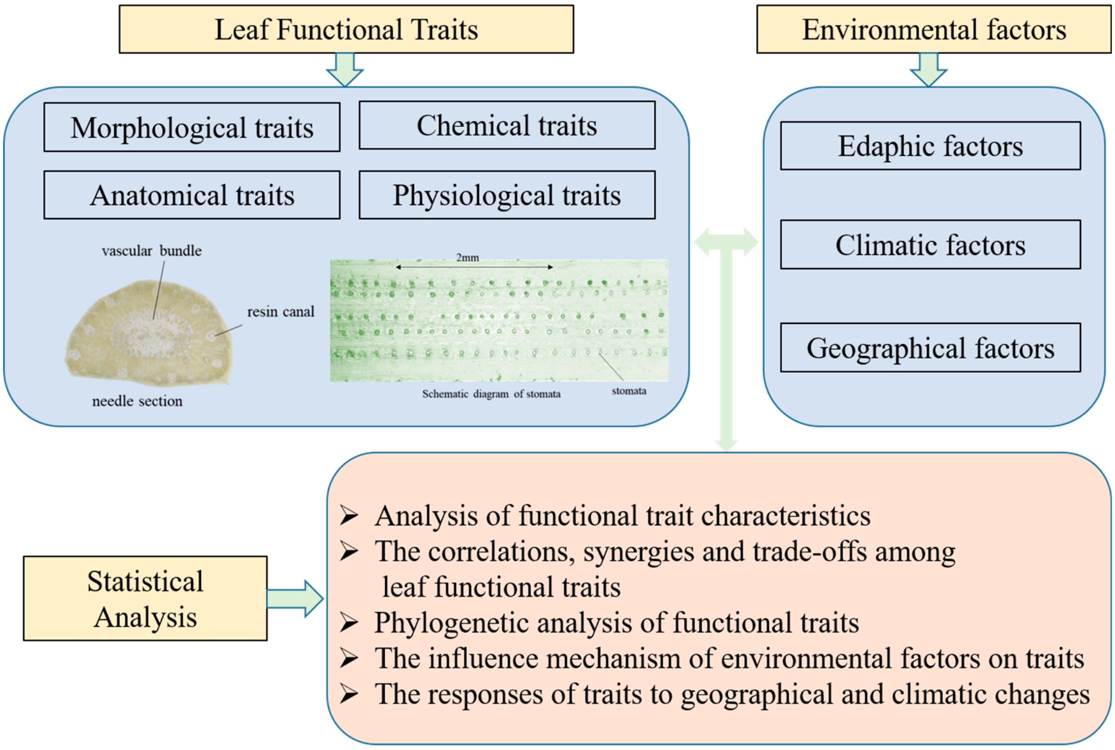

Figure S7 Study the workflow diagram.
